# Combined bioactive PVA nanofibrous scaffolds loaded with Zn ascorbate MOFs and *Petroselinum crispum* (Parsley) extract for enhanced in-vitro and in-vivo wound healing applications

**DOI:** 10.1186/s13036-026-00736-8

**Published:** 2026-08-17

**Authors:** Shimaa Husien, Rana R. Haikal, Eman A. Khalil, Noha El. Salakawy, Ahmed Abdellatif, Wael Mamdouh

**Affiliations:** 1 Department of Chemistry, School of Sciences and Engineering (SSE), The American University in Cairo (AUC), AUC Avenue, P.O. Box 74, New Cairo, 11835 Egypt; 2 Department of Biology, School of Sciences and Engineering, The American University in Cairo (AUC), P.O. Box 74, AUC Avenue, New Cairo, 11835 Egypt

**Keywords:** Nanofibers, Parsley, MOF, Scaffolds, Wound healing, Antibacterial

## Abstract

**Abstract:**

Wounds damage the skin, mucosal membranes, or underlying tissues, disrupting normal structure and function. Natural therapeutic agents, particularly plant extracts, have been widely used in wound management due to their antimicrobial and regenerative properties. Here, we report a novel multifunctional wound dressing by integrating *Petroselinum crispum* (parsley, PS) extract and zinc ascorbate-based metal-organic frameworks (MOFs) into a polyvinyl alcohol (PVA) nanofibrous matrix. The resulting MOF/PS-loaded PVA nanofibers (NFs) combine biodegradability, biocompatibility, and enhanced bioactivity for wound healing. The optimized formulation (PVA/MOF5/PS30-NFs) exhibited strong antibacterial activity, with inhibition zones of ~ 250 mm², total phenolic content of 1.41 mg GAE/g, and antioxidant IC₅₀ values of 15.17 and 11.11 mg/3mL at 2 and 24 h, respectively. Cytocompatibility tests confirmed excellent cell viability (> 100%). In-vitro assays demonstrated enhanced fibroblast migration and edge contraction, with substantial wound closure after 48 h. In-vivo studies showed complete (> 100%) wound closure by day 15, surpassing controls (~ 80%), with the fastest healing trajectory and early acceleration phase observed. To our knowledge, this is the first report of this composition and application. These findings highlight MOF/PS-loaded PVA nanofibrous mats as a promising platform for advanced wound-healing therapies.

**Clinical trial registration:**

Not applicable.

**Graphical Abstract:**

**Figure:**
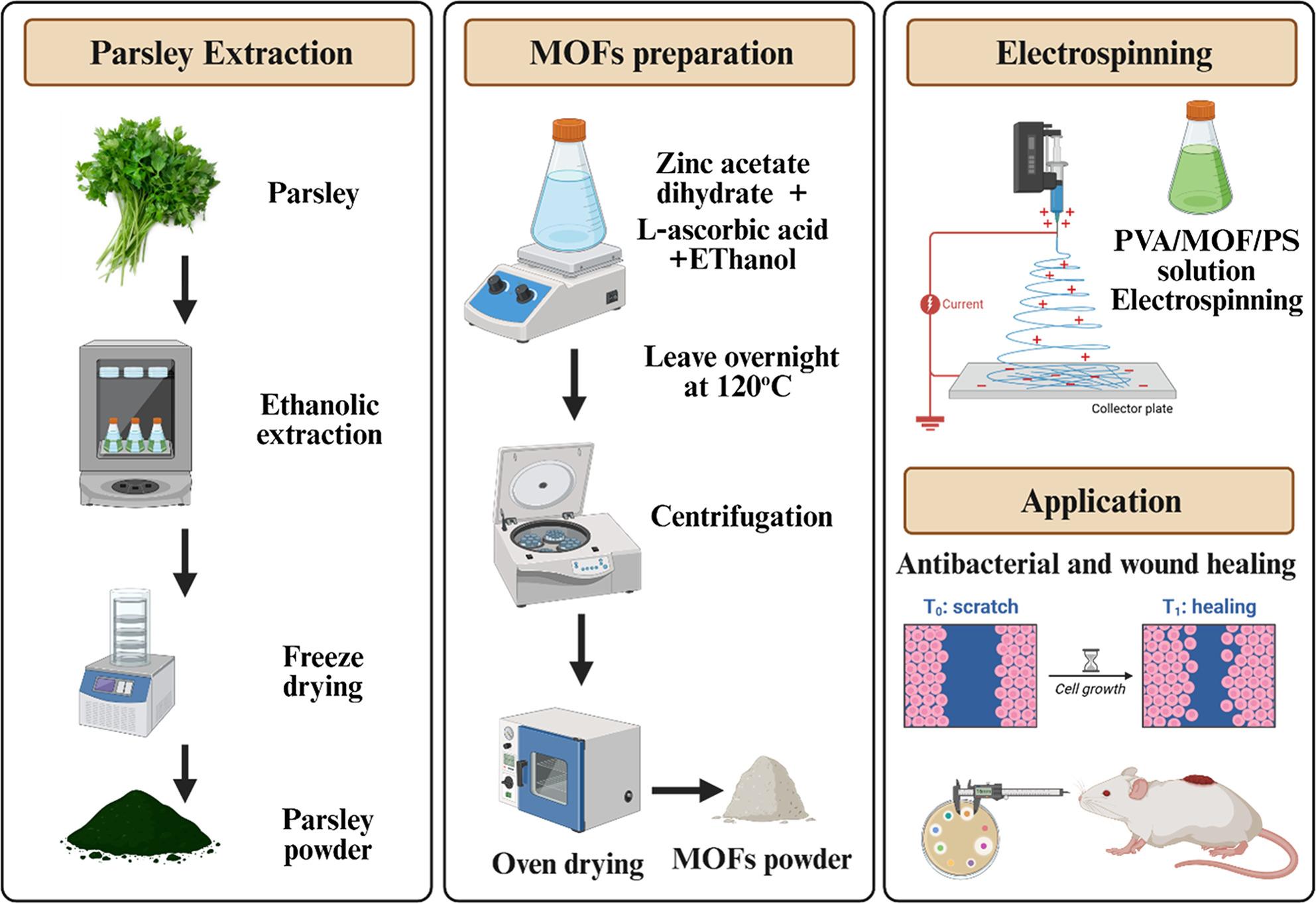

**Supplementary Information:**

The online version contains supplementary material available at 10.1186/s13036-026-00736-8.

## Introduction

Wound healing is a complex and highly orchestrated biological process involving multiple cellular and molecular interactions that repair damaged tissue, promote re-epithelialization, and restore functional integrity [1]. The healing process varies according to wound severity and is generally categorized as acute or chronic. Acute wounds, such as burns or surgical incisions, typically heal rapidly under proper care. In contrast, chronic wounds, including diabetic foot ulcers and pressure ulcers, persist for six weeks or longer and often require early intervention for proper management [2, 3]. The wound-healing process occurs in overlapping yet distinct stages, hemostasis, inflammation, proliferation, angiogenesis, and remodeling. During inflammation, which typically lasts two to three weeks, neutrophils, macrophages, and lymphocytes migrate to the wound site, producing inflammatory mediators like prostaglandins and histamine that enhance vascular permeability and promote immune response [4]. This phase is followed by the proliferative stage, during which new granulation tissue forms, re-epithelialization occurs, and vascular networks are reestablished [5]. The final remodeling stage begins around three weeks post-injury and may continue for several months or years, focusing on restoring tissue tensile strength and normal architecture [6]. Effective wound management requires debridement, infection control, ischemia management, and adequate nutritional support [7]. Traditional therapies have long incorporated natural agents such as honey, propolis, and herbal extracts for their wound-healing potential. In several regions of Africa, Asia, and Latin America, unconventional approaches like leech therapy have also been applied to aid debridement and tissue recovery [8–10]. Modern clinical practices employ various wound dressings, based on materials like collagen, cellulose, hyaluronic acid, and silica gel, to prevent infection, maintain hydration, and facilitate regeneration [11]. Pharmacologic agents including growth factors, corticosteroids, chemotherapeutics, and nonsteroidal anti-inflammatory drugs have also been used for symptom control and microbial inhibition [12]. However, these agents often cause side effects such as immunosuppression, reduced collagen synthesis, and delayed wound repair [13]. Therefore, integrating natural therapeutics into advanced biomaterial-based wound dressing systems represents a promising strategy for improving wound healing outcomes [14–18]. Moreover, the incorporation of biocompatible and biodegradable polymers such as PVA, PCL allows the fabrication of flexible, moist, and breathable wound dressings that mimic the extracellular matrix and enhance healing efficiency [19–21]. Parsley (PS), a widely used medicinal plant, has attracted considerable attention due to its potent antioxidants, anti-inflammatory, and antibacterial properties, making it a promising bioactive agent for wound-healing applications. In recent years, metal-organic frameworks (MOFs), known to be hybrid materials composed of metal ions coordinated with organic ligands, have gained significant attention in biomedical applications [22–24]. Their unique porous structure, high surface area, tunable pore size, and facile surface modification make them ideal platforms for drug encapsulation and controlled release [11]. Certain MOFs also exhibit intrinsic antibacterial properties due to the gradual release of metal ions such as silver, zinc, copper, and cobalt [25, 26].

Recent advances in MOF-polymer wound dressings have demonstrated considerable potential for enhancing antibacterial activity and promoting tissue regeneration [27–29]. However, the integration of naturally derived phytotherapeutics into Zn-ascorbate MOF-based electrospun nanofibrous systems remains largely unexplored. Therefore, this study reports, for the first time, a biodegradable and biocompatible wound dressing composed of parsley (PS) extract-loaded Zn-ascorbate MOFs incorporated into electrospun PVA nanofibers. By combining the antimicrobial activity of Zn-ascorbate MOFs with the antioxidant and anti-inflammatory properties of PS extract within a biomimetic nanofibrous matrix, the developed platform is intended to simultaneously prevent infection, reduce oxidative stress, and support tissue regeneration.

## Materials and methods

### Materials

Zinc acetate dihydrate (98%) and L-ascorbic acid were purchased from Loba Chemie and Research Lab Fine Chem Industries (India), respectively. PVA (average molecular weight ≈ 125,000 g/mol) was obtained from Sigma-Aldrich (USA). Fresh PS was collected from local markets in Egypt. The antioxidant assay reagent 2,2-diphenyl-1-picrylhydrazyl (DPPH) and dichloromethane were supplied by Sigma-Aldrich Co. (USA). Folin-Ciocalteu reagent and gallic acid reference standards were procured from Merck Chemicals (Germany). The MTT cell viability assay reagent (3-(4,5-dimethylthiazol-2-yl)-2,5-diphenyltetrazolium bromide) was obtained from Serva Electrophoresis (Heidelberg, Germany). L929 mouse fibroblast cells (ATCC CCL-1) were sourced from the American Type Culture Collection (USA). All chemicals and reagents were used as received without further purification. All experimental protocols were conducted in compliance with national ethical guidelines and followed the principles outlined in the Guide for the Care and Use of Laboratory Animals.

### Zinc ascorbate-based MOF preparation

Zinc ascorbate-based metal-organic framework (MOF) nanoparticles were synthesized according to the method reported by Haikal et al. [30]. Briefly, zinc acetate dihydrate (1.5 g, 6.8 mmol) and L-ascorbic acid (600 mg, 3.4 mmol) were dissolved in 20 mL of ethanol in a tightly sealed glass vial and stirred until a homogeneous solution was obtained. The synthesis was performed under solvothermal conditions in the sealed vial, which enabled the reaction to proceed at temperatures above the normal boiling point of ethanol through the generation of autogenous pressure while preventing solvent evaporation. The reaction mixture was magnetically stirred at 500 rpm and maintained at 120 °C overnight under atmospheric conditions to facilitate metal-ligand coordination and framework formation. After completion of the reaction, the mixture was allowed to cool naturally to room temperature. The resulting product was collected by centrifugation at 7000 rpm for 5 min, and the supernatant was discarded. The nanoparticles were then washed by resuspension in fresh ethanol followed by centrifugation. This washing procedure was repeated for a minimum of three cycles to ensure the removal of unreacted precursors and residual impurities. The purified product was subsequently dried in an oven at 90 °C and stored under appropriate conditions until further characterization and application studies. The particle yield % is calculated to be around 68% based on the previously reported formula of Zn_3_(Asc)(OH) for the synthesized zinc-ascorbate MOF [30].

### Parsley (PS) extract preparation

The bioactive compounds of PS were extracted using an ethanol-based method. Fresh PS leaves were procured from local Egyptian markets, thoroughly washed to remove surface impurities, and oven-dried at 60 °C until complete dehydration. The dried material was subsequently milled into a fine, homogeneous powder and extracted with 70% (v/v) ethanol at a solid-to-solvent ratio of 1 g per 10 mL of solution at 200 rpm shaking. The extraction conditions were selected based on previous studies reporting that aqueous ethanol solutions at moderate temperatures effectively enhance the recovery of phenolic and flavonoid compounds by improving solvent penetration and mass-transfer efficiency. Although prolonged heating may affect certain thermolabile constituents, extraction at approximately 60 °C has been shown to provide a favorable balance between extraction yield and preservation of bioactive compounds [31, 32]. The suspension was incubated on a shaker at approximately 60 °C for 24 h to facilitate efficient extraction. After incubation, the mixture was filtered, and the obtained filtrate was concentrated using a rotary evaporator operated at 65 °C and 200 rpm until the solvent was entirely evaporated [33]. The concentrated ethanolic extract was then refrigerated for subsequent characterization and experimental use. Following freeze-drying, the extraction yield was determined, and the concentration of the obtained PS was calculated to be 8 mg/mL with approximately 8% yield. In addition, a calibration curve was established for PS, and its characteristic absorption maximum was determined using UV–Vis spectrophotometry, with a λmax observed at approximately 340 nm.

### GC-MS analysis of the parsley (PS) extract

A portion of the PS ethanolic extract was frozen and subsequently lyophilized using a freeze dryer (BK-FD10S, Biobase Biodustry Co., Ltd., China) prior to gas chromatography-mass spectrometry (GC-MS) analysis. The lyophilized extract was reconstituted to a concentration of 10 mg/mL and filtered through a 0.45 μm membrane filter (Millipore, USA) before analysis. Subsequently, 1 µL of the filtered sample was injected into the GC–MS system. Trace GC 1310 gas chromatograph coupled with an ISQ mass spectrometer (Thermo Scientific, Austin, TX, USA) equipped with a TG-5MS capillary column (30 m × 0.25 mm i.d., 0.25 μm film thickness) was used for the analysis process. The oven temperature was initially set at 35 °C and increased at a rate of 3 °C/min to 200 °C, where it was held for 3 min. The temperature then further increased to 280 °C at the same heating rate and maintained for an additional 10 min. The injector and transfer line temperatures were maintained at 250 °C and 260 °C, respectively. Helium was used as the carrier gas at a constant flow rate of 1 mL/min. Prior to analysis, the samples were appropriately diluted, and a 1 µL aliquot was injected using an AS1300 autosampler operating in spitless mode with a solvent delay of 3 min. Mass spectra were acquired under electron ionization (EI) conditions at 70 eV in full-scan mode over an m/z range of 40-1000. The ion source temperature was maintained at 200 °C. Identification of the detected compounds was achieved by comparing the obtained mass spectra and retention indices with those available in the WILEY 09 and NIST 11 mass spectral libraries [34, 35].

### Nanofibers electrospinning and optimization

NF precursor solutions were prepared using 10% (w/v) PVA. Specifically, 1 g of PVA powder (Mw = 125,000 g/mol) was dissolved in 10 mL of distilled water in a tightly sealed vessel and stirred at 120 °C for 2 h until complete dissolution. For PVA/MOF NFs, the required amount of MOF nanoparticles was initially dispersed in distilled water using probe sonication (model: Qsonic, sonication, USA) for 2 min in a 2 s on/2 s off pulse mode at 50% amplitude. The dispersion was carried out in an ice bath to minimize temperature rise induced by sonication. The MOF suspension was sonicated to obtain a homogeneous and stable dispersion, which was subsequently used as the solvent medium for dissolving PVA. After cooling, the PS extract was incorporated into the PVA/MOF precursor blend at different volume-to-volume ratios, as summarized in Table 1. Optimization experiments were designed by varying the MOF content to 1.25%, 2.5%, and 5% (w/w, relative to PVA), along with PS extract volumes of 10%, 20%, 30%, and 40% (v/v, extract to solution). The best-performing MOF concentration was subsequently combined with each PS level to fabricate composite PVA/MOF/PS NFs. The NFs’ morphology, diameter uniformity, antioxidant capacity, and antibacterial performance were assessed as response variables to determine the optimal formulation parameters. Electrospinning was performed using a custom-built apparatus with a stationary flat-plate collector operated at a flow rate of 0.5 mL/h, a tip-to-collector distance of 16 cm, and an applied voltage of 21 kV. A 10 mL medical syringe fitted with a stainless-steel needle (gauge: 21G) was used as the spinneret. The experiments were performed under ambient laboratory winter conditions in Egypt, with a temperature of approximately 18–22 °C and a relative humidity of 40–55%. For each formulation, a total volume of 10 mL of polymer solution was electrospun. All processing parameters were kept constant across all samples to ensure reproducibility and reliable comparison between formulations.

**Table 1:** Applied parameters for the PVA/MOF/PS NFs optimization process

Parameters	Unit	Levels			
MOF Conc. in PVA/MOF-NFs	**(mg/mL)**	1.25	2.5	5	-
PS Conc. in PVA/PS-NFs	**(% v/ v)**	10	20	30	40
PS Conc. in PVA/MOF/PS-NFs	**(% v/v)**	10	20	30	40
Responses	Diameter/Morphology				
	Antioxidant activity				
	Antibacterial activity				

### Scanning Electron Microscope (SEM) imaging analysis

The surface morphology and NF diameter of the fabricated NFs were characterized using field emission scanning electron microscopy (FE-SEM, Leo Supra 55; Zeiss Inc., Germany). Prior to imaging, the samples were sputter-coated with a thin layer of gold at 10 mA for 6 min to enhance conductivity. SEM micrographs were acquired at magnifications of ×2000 and ×10,000. The average fiber diameter was calculated by measuring approximately 100 randomly selected fibers from each sample using ImageJ software, while the diameter distribution was statistically analyzed with Origin^®^ 2025. The optimal nanofiber formulation was identified based on the smallest average fiber diameter, absence of bead formation, and uniform fiber morphology. Additionally, the morphology and microscopic size of the optimum PVA/MOF5/PS30-NFs were examined using Transmission Electron Microscopy (TEM) (JEOL JEM, Japan).

### Antibacterial activity

Antibacterial activity was evaluated as one of the key response parameters to determine the optimum concentrations of MOF NPs and PS extract incorporated within the nanofiber formulations. The antibacterial performance of PVA-NFs, PVA/MOF-NFs, PVA/PS-NFs, and PVA/MOF/PS-NFs at various concentrations was assessed using the disk diffusion method as described by Khalid et al. Mueller-Hinton agar plates were inoculated with 100 µL of bacterial suspension containing approximately 1–3 × 10⁵ CFU/mL [36]. The test microorganisms included *Escherichia coli* (ATCC 25922) (*E. coli*) as a Gram-negative model and *Staphylococcus aureus* (ATCC 25923) (*S. aureus*) as a Gram-positive strain. Nanofiber mats were cut into 1 × 1 cm² pieces (Approximately 5 mg weight for each sample). Prior to testing, the samples were sterilized by ultraviolet (UV) irradiation at a wavelength of 254 nm for 15 min on each side. Both surfaces were exposed to UV light to ensure effective sterilization, after which the samples were aseptically placed onto the inoculated agar plates. The bacterial inoculum was adjusted to a final concentration of 3 × 10⁵ CFU/mL and standardized using the McFarland turbidity standard prior to use. The plates were incubated at 37 °C for 24 h, and the diameters of the inhibition were measured to quantify antibacterial efficacy. All experiments were performed in triplicate, and results were expressed as mean ± standard deviation.

### Antioxidant activity/IC_50_

The antioxidant activity of all fabricated nanofibers, in addition to the PS extract, was evaluated using the DPPH radical scavenging assay to determine the optimal MOF/PS concentrations for incorporation into the nanofibers. A 0.05 mM DPPH solution was prepared, and all samples (8 mg of the prepared NFs in 3 mL DPPH solution) were incubated in the dark for 2 and 24 h. The samples were then centrifuged at 7500 rpm for 3 min prior to analysis. The absorbance of the resulting supernatants was then measured spectrophotometrically at 517 nm using a UV-Vis spectrophotometer (Varian Cary 500, Agilent Technologies, USA). The percentage of DPPH inhibition was calculated based on **Eq. 1**. After that the selected optimum fabricated NFs were investigated with different weights to determine the IC_50_ values corresponding to 50% DPPH inhibition.

1 $$\:Antioxidant\:\left(\%\right)=\:\frac{Acontrol-Asample}{Acontrol}\:\times\:100$$

where Acontrol is the absorbance of the DPPH reagent, A sampleis the absorbance of the prepared samples.

### Optimum nanofibers characterization

The optimized NF formulations, PVA-NFs, PVA/MOF5-NFs, PVA/PS30-NFs, and PVA/MOF5/PS30-NFs, were selected for further analysis. These samples were subsequently characterized using a range of physicochemical and functional evaluation techniques. Morphological features, including fiber diameter and surface structure, as well as antibacterial activity, were examined. Comprehensive characterization was carried out using Fourier Transform Infrared (FT-IR) Spectroscopy, Thermogravimetric Analysis (TGA), X-ray Diffraction (XRD), tensile strength testing, swelling and solubility measurements, total phenolic content (TPC) determination, and antioxidant activity assessment. The experimental procedures for each technique were conducted as described below:

#### Fourier Transform-Infrared (FT-IR) Spectroscopy

The chemical composition and functional groups of the optimized NFs, PVA-NFs, PVA/MOF5-NFs, PVA/PS30-NFs, and PVA/MOF5/PS30-NFs, were examined using FT-IR Spectroscopy (Thermo Scientific Nicolet 8700, USA). For powdered samples, spectra were obtained using the KBr pellet technique, while intact NF mats were analyzed directly. All spectra were recorded within the wavenumber range of 4000–400 cm⁻¹.

#### Thermogravimetric analysis (TGA)

The thermal stability of the optimized NFs, PVA-NFs, PVA/MOF5-NFs, PVA/PS30-NFs, and PVA/MOF5/PS30-NFs, was evaluated using TGA (Q50 Thermogravimetric Analyzer, TA, USA). Approximately 5 mg of each sample was placed in an aluminum crucible and heated from room temperature to 600 °C at a rate of 10 °C/min over a total duration of 76 min. Differential thermogravimetric (DTG) curves were obtained to determine the rate of weight loss (da/dt) as a function of temperature. The resulting TGA and DTG profiles were analyzed and discussed in the *Results and Discussion* section.

#### X-Ray Diffraction (XRD)

X-ray diffraction (XRD) analysis was performed using an Advanced X-ray Solutions diffractometer to examine the crystalline structure of the prepared NFs, including PVA-NFs, PVA/MOF5-NFs, PVA/PS30-NFs, and PVA/MOF5/PS30-NFs composites. The measurements were carried out using Cu Kα radiation equipped with a Ni filter, operated at 40 kV and 30 mA. Mechanical properties were subsequently evaluated using a universal testing machine (Instron 5569 Load Frame, USA) fitted with a 5 kN load cell to determine the tensile performance of the nanofiber mats.

#### Tensile strength

The optimized nanofiber (NF) formulations, namely PVA NFs, PVA/MOF5 NFs, PVA/PS30 NFs, and PVA/MOF5/PS30 NFs, were fabricated and subsequently subjected to mechanical characterization. Each NF mat was cut into dumbbell-shaped specimens measuring 36 × 8 mm in accordance with the ASTM D882 standard, maintaining a gauge length of 10 mm. The tensile strength of the samples was measured using In-Situ Specimen Tensile Stage instrument (TSL Solutions Co., Ltd., Japan) with a maximum load capacity of 1500 N. After that, the data obtained were plotted and statistically analyzed.

#### Swelling and solubility

The swelling and solubility behaviors of the optimized NF mats, PVA-NFs, PVA/MOF5-NFs, PVA/PS30-NFs, and PVA/MOF5/PS30-NFs, were assessed using phosphate-buffered saline (PBS) over a 24 h period. The dry membranes (1 × 1 cm) were first accurately weighed to determine their initial weight (W₁), then immersed in PBS. After 24 h, the samples were removed, gently blotted to remove surface moisture, and weighed again to record the swollen weight (W₂). The membranes were subsequently oven-dried at 60 °C until constant weight was achieved, and the final dry weight (W₃) was measured. The swelling ratio and solubility percentage were calculated according to **Eqs. (2) and (3)**, respectively, following the method described by reference. The results obtained were plotted and discussed in the *Results and Discussion* section.

2 $$\:Swelling\:\left(\%\right)=\frac{{W}_{2}-{W}_{1}}{{W}_{1}}*100$$

3 $$\:Solubility\:\left(\%\right)=\frac{{W}_{1}-{W}_{3}}{{W}_{1}}*100$$

#### Total Phenolic content (TPC)

The total phenolic content (TPC) of the NF samples, PVA-NFs, PVA/MOF5-NFs, PVA/PS30-NFs, and PVA/MOF5/PS30-NFs, was quantified using the Folin-Ciocalteu method. Each NF sample was dissolved in ethanol to prepare a 1 mg/mL solution. Subsequently, 0.5 mL of the sample solution was mixed with 2.5 mL of 10% (v/v) Folin-Ciocalteu reagent, followed by the addition of 2 mL of 7.5% (w/v) sodium carbonate solution. The reaction mixtures were incubated in the dark for 6 h to facilitate color development. After incubation, the solutions were centrifuged at 7500 rpm for 3 min, and absorbance was measured at 765 nm using a UV–Vis spectrophotometer. TPC values were calculated from a gallic acid calibration curve and expressed as milligrams of gallic acid equivalents per gram of dry sample (mg GAE/g) [37].

### Release profiles and kinetics

The controlled release profiles of PS extract from the optimized PVA/PS30-NFs, and PVA/MOF5/PS30-NFs, were evaluated in PBS (pH 7.4). For each experiment, 25 mg of dried NFs were sealed within a dialysis membrane (molecular weight cut-off: 12,000–14,000 Da) containing 3 mL of PBS. This dialysis bag was then immersed in 12 mL of the same release medium and maintained at room temperature with gentle stirring. At predetermined time intervals, 1 mL aliquots were withdrawn from the release medium for analysis and immediately replaced with an equal volume of fresh PBS to ensure sink conditions. The concentration of released PS extract in the external medium was quantified by UV–Vis spectrophotometry at approximately 340 nm. The cumulative release percentage was calculated according to the established release kinetics models to

4 $$\:\mathrm{C}\mathrm{u}\mathrm{m}\mathrm{u}\mathrm{l}\mathrm{a}\mathrm{t}\mathrm{i}\mathrm{v}\mathrm{e}\:\mathrm{r}\mathrm{e}\mathrm{l}\mathrm{e}\mathrm{a}\mathrm{s}\mathrm{e}\:\mathrm{\%}={\sum\:}_{\mathrm{t}=0}^{\mathrm{t}}\frac{{\mathrm{M}}_{\mathrm{t}}}{{\mathrm{M}}_{0}}\times\:100\:$$

Where the cumulative quantity of PS extract released at each time interval is ($\:{M}_{t}$), and the initial amount of PS extract in the PVA/PS-NFs, and PVA/MOF/PS-NFs is ($\:{M}_{0}$).

To elucidate the release mechanism, the release data were analyzed by fitting into several kinetic models, including zero-order, first-order, Korsmeyer-Peppas, Higuchi, and Weibull.

### In-vitro and in-vivo wound healing assay

#### In-vitro toxicity (MTT) assay

The in-vitro cytocompatibility of the fabricated NFs, PVA‑NFs, PVA/MOF5‑NFs, PVA/PS30‑NFs, and PVA/MOF5/PS30‑NFs, was assessed using mouse fibroblast L929 cells (ATCC). L929 mouse fibroblast cells were cultured in complete DMEM supplemented with 10% fetal bovine serum and 1% penicillin-streptomycin and maintained at 37 °C in a humidified atmosphere containing 5% CO₂ using a HeraCell CO₂ incubator (Thermo Fisher Scientific, Germany). Cells between passages 10 and 15 were used for all experiments.

The extraction procedure was performed by immersing NF samples (0.5 × 0.5 cm) in 1 mL of complete culture medium (Dulbecco’s Modified Eagle Medium (DMEM) supplemented with 5% fetal bovine serum (FBS) and 1% penicillin-streptomycin) under dynamic conditions using a shaking incubator for 24 h at 37 °C to generate extraction media. The extraction ratio was approximately 0.7 cm²/mL.

The extracts were sterilized through a 0.22 μm syringe filter and added to 96‑well plates containing L929 cells seeded at a density of 10,000 cells/well. Following a 24 h incubation, MTT [3‑(4,5‑dimethylthiazol‑2‑yl) -2,5‑diphenyltetrazolium bromide; Serva Electrophoresis, Heidelberg, Germany] stock solution (5 mg/mL in PBS) was added to fresh culture medium at a ratio of 20:80 (v/v, MTT stock: culture medium). The cells were then incubated for an additional 3 h to allow formazan formation. The MTT solution was then aspirated, and the formed formazan crystals were dissolved in 120 µL of dimethyl sulfoxide (DMSO; Lonza, Belgium). Cell viability was quantified by measuring absorbance at 570 nm using a microplate reader (SPECTRO star Nano, BMG Labtech, Ortenberg, Germany) and cell viability was calculated relative to untreated controls.

#### In-vivo wound healing assessment

#### Animals and surgical procedures

Male Sprague Dawley rats (*Rattus norvegicus domestica*, RRID: MGI:5651135; 150–200 g body weight) were procured from the Theodore Bilharz Research Institute (TBRI) and housed under standard laboratory conditions with *ad libitum* access to food and water. All animal procedures complied with the National Institutes of Health guidelines (NIH Publication No. 85 − 23, Revised 2011) and received approval from the Institutional Animal Care and Use Committee of the American University in Cairo (AUC-IACUC). Anesthesia was induced by intraperitoneal administration of ketamine (80–100 mg/kg) and xylazine (10–12.5 mg/kg). Full-thickness excisional wounds (1 cm diameter) were created on the shaved and Betadine-disinfected dorsal surface of each rat using sterile surgical scissors, following the protocol described by Sami and Abdellatif (2020). Wound healing was monitored photographically by blind observers until complete closure. On day 15, rats were euthanized with an overdose of ketamine and xylazine at the doses, and wound sites, including adjacent skin and muscle tissues, were excised and fixed in 10% neutral buffered formalin for subsequent histological analysis.

#### Histopathological examination

On day 15, rats were euthanized with an overdose of ketamine and xylazine at the doses, and wound sites, including adjacent skin and muscle tissues, were excised and fixed in 10% neutral buffered formalin for subsequent histological analysis. Harvested skin tissues were fixed in 10% formalin saline, trimmed, and processed through dehydration in a graded ethanol series followed by xylene clearing. The cleared samples were embedded in paraffin blocks, sectioned at 4–6 μm thickness, and deparaffinized using xylene. Tissue sections were stained with hematoxylin and eosin (H&E) for general morphology and Masson’s trichrome for collagen visualization, as described by Bancroft et al. [38]. Histopathological evaluation was conducted under light microscopy. A semi-quantitative scoring system was employed to grade key wound healing parameters re-epithelialization, polymorphonuclear leukocyte (PMNL) infiltration, fibroblast proliferation, neovascularization, and collagen deposition—on a scale of 0 to 4, following established protocols [39, 40], See Table**S1**.

## Results and discussion

### GC-MS analysis of the PS extract

Gas chromatography-mass spectrometry (GC–MS) analysis of the PS hydroethanolic extract revealed a complex phytochemical composition comprising diverse fatty acids, aliphatic acids, and long-chain hydrocarbons (Table 2). The predominant constituents were identified as hexadecanoic acid ethyl ester (ethyl palmitate, 6.59%), ethyl (9Z,12Z)-9,12-octadecadienoate (ethyl linoleate, 8.79% and 1.97% in two peaks), and 9-octadecenoic acid ethyl ester (ethyl oleate, 16.97%). Additional long-chain saturated and unsaturated fatty acid esters such as docosanoic acid ethyl ester (ethyl behenate, 1.29%), eicosanoic acid ethyl ester (ethyl arachidate, 1.10%), and trans-9-octadecenoic acid pentyl ester (1.69%) were also identified. Minor components included methyl eicosenoate (4.93%), methyl nervonate (2.19%), and ethyl lignocerate (1.05%). The obtained profile aligns with previous GC–MS characterizations of PS extracts and essential oils, which commonly exhibit a predominance of long-chain fatty acids and volatile aromatic compounds. Prior studies have consistently reported hexadecanoic (palmitic) and octadecenoic (oleic) acid derivatives, particularly their ethyl esters, as major constituents contributing to the antimicrobial and antioxidant activities of PS oils and extracts [41, 42]. Other investigations have also detected phenylpropanoids such as myristicin, apiol, and D-limonene as key bioactive compounds [43]. Comparative analyses of PS essential oils and hydroethanolic extracts demonstrate that, while both contain similar classes of bioactive molecules, their chemical profiles vary significantly depending on the extraction method and solvent polarity. Tang et al. (2015) reported that sequential extraction using solvents of increasing polarity (hexane, dichloromethane, ethyl acetate, methanol, and water) yielded extracts with markedly different phenolic contents, with the dichloromethane fraction exhibiting the highest levels, emphasizing the critical role of solvent polarity in the recovery of phenolic compounds [44]. Furthermore, other GC-MS investigations of PS extract have identified phenylpropanoids (e.g., myristicin, apiol, β-phellandrene), fatty acids (e.g., palmitic, oleic, linoleic acids), and phenolic compounds, with variations in relative abundance influenced by extraction technique, plant part, and environmental conditions [45, 46]. The present study underscores the predominance of fatty acid ethyl esters in the hydroethanolic extract, consistent with established lipid-based profiles of PS extract. Although the current analysis did not reveal prominent levels of phenylpropanoids, the extract’s high content of unsaturated fatty acid esters, aromatic ester-bearing molecules, and semi-volatile components suggest potential indirect contributions of phenylpropanoid-type activity. These semi-lipophilic compounds are known to enhance membrane interaction, antioxidant defense, and antimicrobial efficacy through enhanced effects such as modulation of membrane permeability, stabilization of reactive intermediates, and facilitation of the uptake of minor aromatic molecules [47]. Collectively, while free myristicin and apiol were not directly detected, the GC-MS profile indicates a functionally relevant phenylpropanoid signature. This signature is manifested through lipid-associated and aromatic ester constituents, which likely contribute to the overall antioxidant, antimicrobial, and anti-inflammatory activities of the PS hydroethanolic extract.

**Table 2:** GC-MS profile of hydroethanolic PS extract

RT	Peak %	Detected GC-MS Compound	Trivial name
48.96	**6.59**	Hexadecanoic acid, ethyl ester	Ethyl palmitate
53.95	**8.79**	Ethyl (9z,12z)-9,12-octadecadenoate	Ethyl linoleate
54.03	**1.97**	9,12-octadecadenoic acid, ethyl ester	Ethyl linoleate
54.51	**16.97**	9-octadecenoic acid (z)-, ethyl ester	Ethyl oleate
54.61	**1.38**	Ethyl oleate	Ethyl oleate
55.33	**3.85**	Octadecanoic acid, ethyl ester	Ethyl stearate
61.11	**4.93**	Cis-methyl 11-eicosenoate	Methyl gondoate
62.00	**1.12**	Eicosanoic acid, ethyl ester	Ethyl arachidate
67.41	**10.23**	Ethyl 13-docosenoate (ethyl erucate)	Ethyl erucate
67.88	**1.29**	Docosanoic acid, ethyl ester	Ethyl behenate
68.23	**1.10**	Eicosanoic acid, ethyl ester	Ethyl arachidate
68.44	**1.69**	Trans-9-octadecenoic acid, pentyl ester	Pentyl elaidate
72.13	**2.19**	15-tetracosenoic acid, methyl ester, (z)-	Methyl nervonate
72.80	**1.05**	Ethyl tetracosanoate	Ethyl lignocerate
87.97	**3.11**	9-hexadecenoic acid, 9-hexadecenyl ester, (z)-	Palmitoleyl palmitoleate
89.06	**1.26**	9-octadecenoic acid (z)-	Oleic acid

### Electrospinning optimization

The concentrations of PS extract and MOF NPs were systematically optimized by varying their contents within the PVA-NFs matrix. Three MOF loadings (1.25, 2.5, and 5%) and four PS extract concentrations (10, 20, 30, and 40%) were examined to identify the optimal combination yielding smooth, bead-free NFs with minimal average diameter and enhanced antioxidant and antibacterial activity. The morphologies of the electrospun NFs prepared under different MOF, PS, and MOF/PS concentration conditions are presented in Figures**S1****-****S1****1**, while Table 3 summarizes the corresponding fiber diameters for each formulation. SEM analysis revealed that increasing the MOF concentration to 5% resulted in uniform, bead-free fibers with the lowest mean diameter of 114.5 nm. Similarly, NFs containing 30% PS exhibited a consistent and homogeneous morphology without bead formation, with an average fiber diameter of 180.2 nm. The combination of these two optimized components, 5% MOF and 30% PS, produced the most favorable formulation, designated as PVA/MOF5/PS30-NFs, characterized by uniform, bead-free fibers with an average diameter of approximately 167.6 nm. Optimization of the MOF and PS concentrations was guided not only by the morphological characteristics but also by the antioxidant and antibacterial performance of the resulting NFs.

**Fig. 1:**
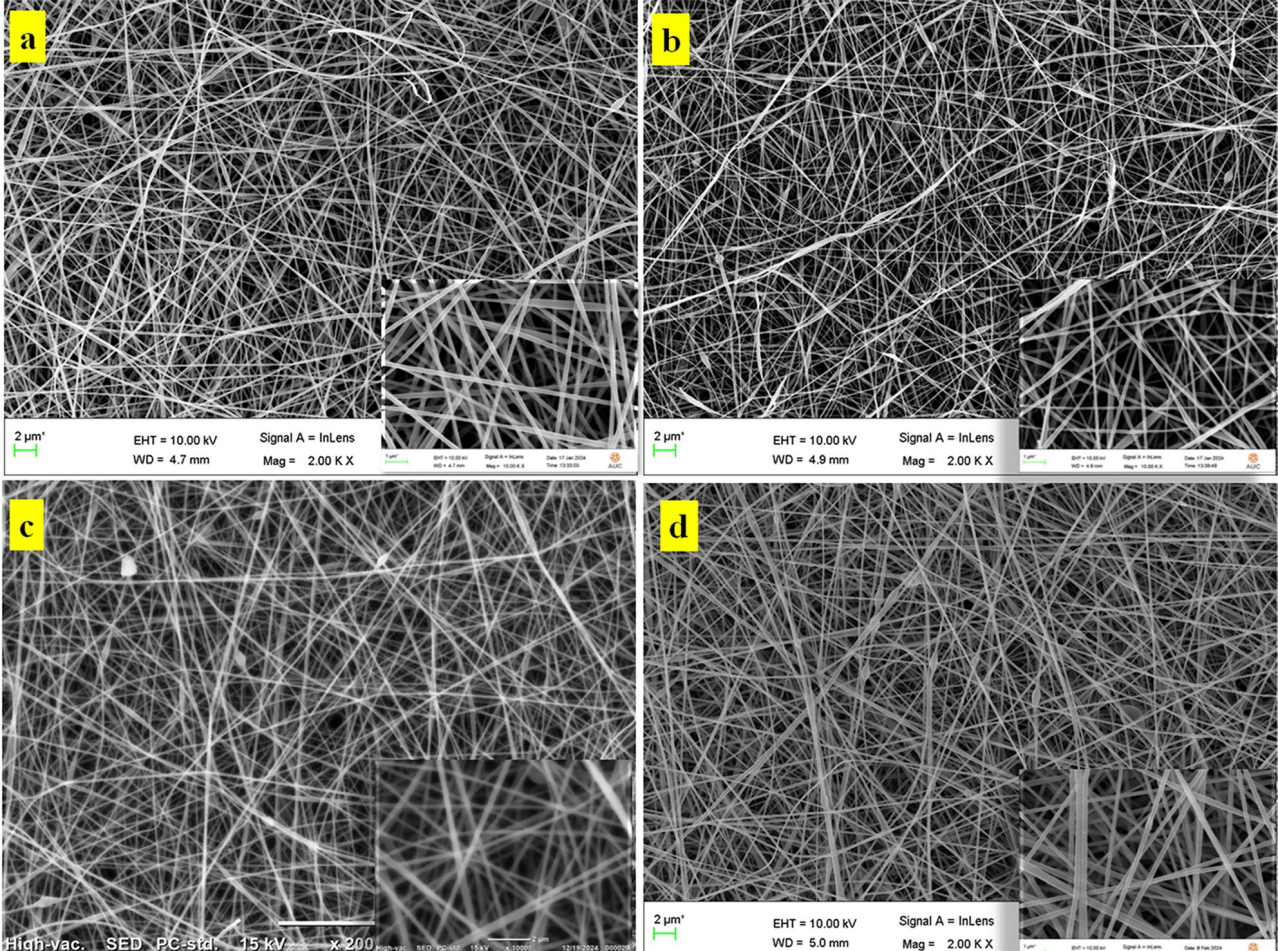
SEM images of the optimum prepared nanofibers (**a**) PVA-NFs, (**b**) PVA/MOF5-NFs, (**c**) PVA/PS30-NFs, (**d**) and PVA/MOF5/PS30-NFs

The optimum SEM micrographs shown in Fig. 1 illustrate uniform, bead-free NF structures with average diameters of 168.5, 114.5, 180.2, and 167.7 nm for PVA-NFs, PVA/MOF5-NFs, PVA/PS30-NFs, and PVA/MOF5/PS30-NFs, respectively. It was observed that, incorporation of MOF into PVA-NFs significantly reduced the fiber diameter from 168.5 nm to 114.5 nm. This could be attributed to enhanced solution conductivity and increased charge density during electrospinning because of MOF addition, which promotes greater jet stretching and thinning under the applied electric field [48, 49]. This result is consistent with observations in Zn-MOF/PVA systems where inorganic particles facilitate finer fiber formation by disrupting polymer chain entanglement and reducing solution viscosity [50]. In contrast, loading with PS extract increased the diameter to 180.2 nm, attributable to the elevated solution viscosity induced by the polyphenolic and macromolecular components of the hydroethanolic extract, which resist jet elongation and favor thicker fibers, as demonstrated in polyphenol-rich plant extract/PVA composites where extract addition proportionally enlarges nanofiber diameters [51]. Notably, the combined PVA/MOF5/PS30-NFs formulation yielded a diameter (167.7 nm) nearly identical to plain PVA-NFs (168.5 nm), suggesting a compensatory interaction where the diameter-reducing conductivity effects of MOF offset the thickening viscosity influence of PS, resulting in balanced electrospinning dynamics akin to combined MOF/organic additive systems that maintain morphology close to the polymer baseline.

**Table 3:** The diameter of the prepared nanofibers determines by Image J software

NFs-formulations	PVA Conc. (%wt./v)	MOF Conc. (mg/mL)	PE Conc. (%v/v)	Diameter (nm)	NFs description
*PVA-NFs*	***10***	***-***	***-***	***168.5***	***Uniform with no beads***
PVA/MOF1.25-NFs	10	1.25	-	144.9	***Uniform with no beads***
PVA/MOF2.5-NFs	10	2.5	-	149.5	***Uniform with no beads***
*PVA/MOF5-NFs*	***10***	***5***	***-***	***114.5***	***Uniform with no beads***
PVA/PE10-NFs	10	-	10	169.8	***Not Uniform with no beads***
PVA/PE20-NFs	10	-	20	202.7	***Uniform with beads***
*PVA/PE30-NFs*	***10***	***-***	***30***	***180.2***	***Uniform with minor beads***
PVA/PE40-NFs	10	-	40	177.7	***Uniform with beads***
PVA/MOF5/PE10-NFs	10	5	10	191.8	***Uniform with minor beads***
PVA/MOF5/PE20-NFs	10	5	20	161.9	***Uniform with minor beads***
*PVA/MOF5/PE30-NFs*	***10***	***5***	***30***	***167.7***	***Uniform with minor beads***
PVA/MOF5/PE40-NFs	10	5	40	163.6	***Uniform with minor beads***

### Biological activity-guided optimization of nanofiber formulation

**Fig. 2:**
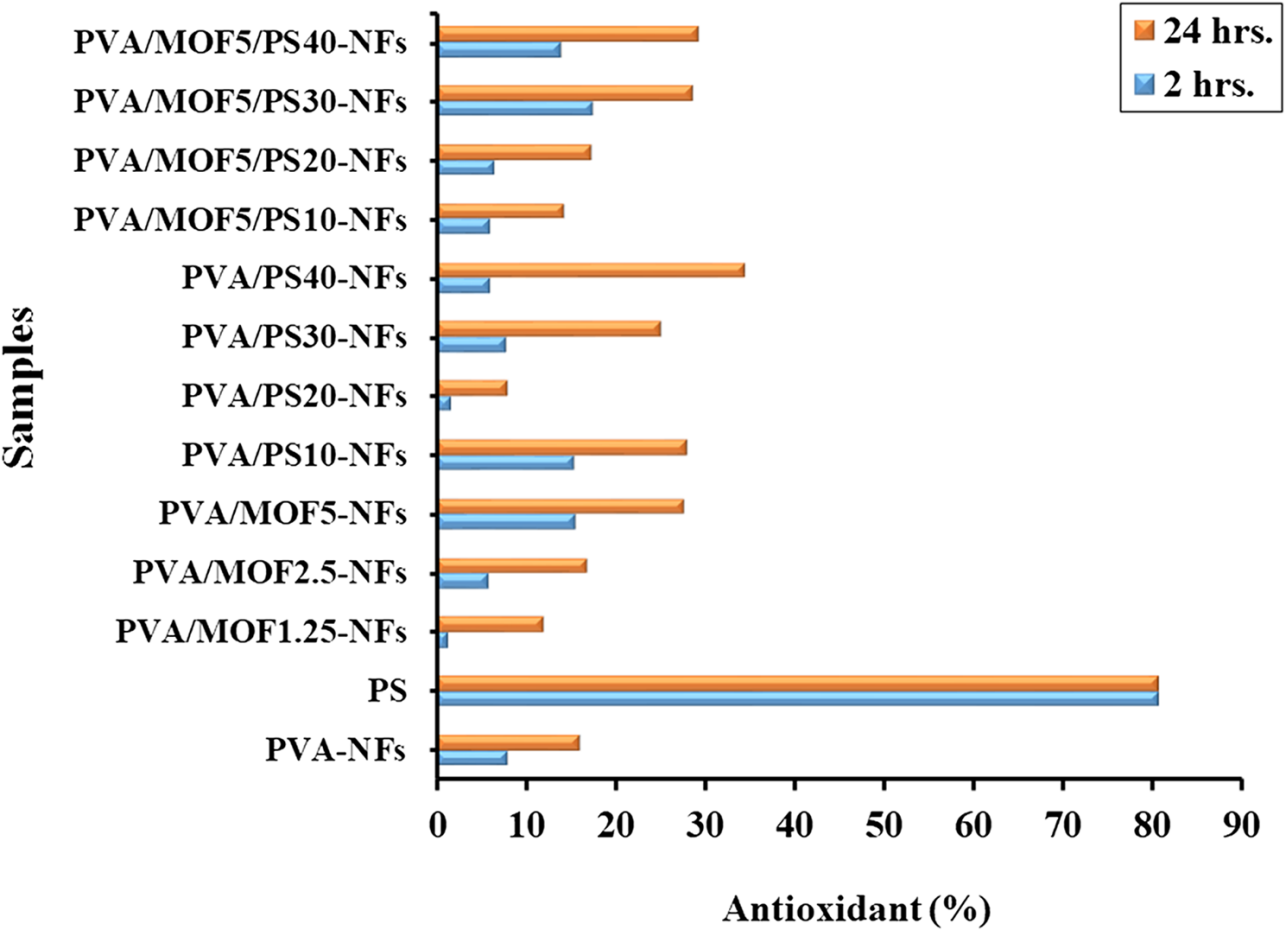
Antioxidant activity of the various prepared nanofibers

The selection of the optimal NF formulation was further guided by evaluating its antioxidant and antibacterial activities. The antioxidant activity of the NFs composites (Fig. 2) revealed distinct trends across formulations. PVA/MOF-NFs exhibited enhanced antioxidant activity with increasing MOF NPs content. Specifically, PVA/MOF1.25-NFs, PVA/MOF2.5-NFs, and PVA/MOF5-NFs achieved radical scavenging percentages of approximately 8%, 16%, and 28% after 24 h, respectively, compared to lower values (~ 1–15%) after 2 h, indicating time-dependent radical scavenging. In contrast, PVA/PS-NFs showed activity that rose with both PS content and incubation time; for example, PVA/PS10-NFs and PVA/PS40-NFs recorded ~ 25% and ~ 42% after 24 h versus ~ 12% to ~ 8% after 2 h, respectively, underscoring sustained release of antioxidant components from the PS-loaded nanofibrous matrices. Neat PVA-NFs displayed minimal activity (~ 12% after 24 h), confirming MOF NPs and PS’s pivotal roles in radical scavenging. PVA/MOF/PS-NFs indeed outperformed all other composites due to combined MOF-PS integration. PVA/MOF5/PS30-NFs and PVA/MOF5/PS40-NFs reached nearly 30% after 2 h and over 35% after 24 h, while intermediate formulations (e.g., PVA/MOF5/PS10-NFs, PVA/MOF5/PS20-NFs) exhibited proportionally lower activity, reflecting a direct correlation with PS content. These findings demonstrate that the PVA/MOF5/PS30-NFs formulation offers a promising strategy for sustained antioxidant activity along with uniform morphological structure, suitable for biomedical and food packaging applications.

**Fig. 3:**
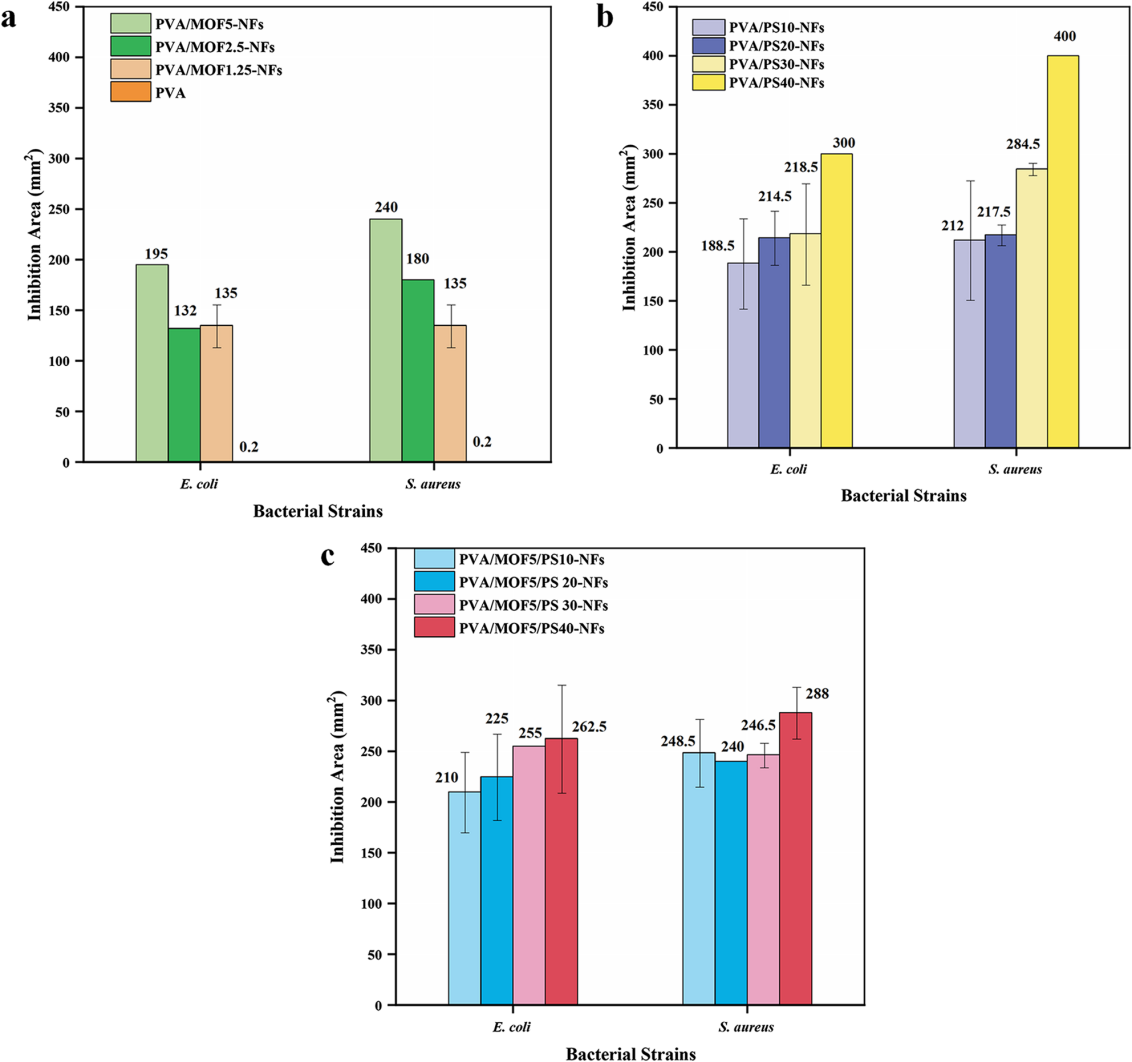
Antibacterial activity of the different prepared NFs

The composites NFs’ antibacterial properties (Fig. 3) were more effective against *Staphylococcus aureus (S. aureus)* compared to *Escherichia coli* (*E. coli*). Figure 3a shows the PVA/MOF-NFs systems, which exhibited a pronounced antibacterial activity against both *E. coli* and *S. aureus*, with inhibition areas increasing as the MOF NPs loading increased, whereas pristine PVA showed only insignificant zones of inhibition for both strains. PVA/MOF1.25-NFs produced inhibition areas of approximately 120–130 mm² against *E. coli* and 150–160 mm² against *S. aureus*, respectively, while PVA/MOF2.5-NFs reached about 150–160 mm² and 200–210 mm², respectively. PVA/MOF5-NFs demonstrated the highest antibacterial performance, with inhibition zones close to 180–190 mm² for *E. coli* and 230–240 mm² for *S. aureus*. The enhanced antibacterial efficacy can be attributed to the intrinsic antimicrobial properties of zinc ascorbate-based MOFs, which arise from the release of zinc and ascorbate ions and the generation of reactive oxygen species (ROS), leading to disruption of bacterial cell membranes and intracellular components [52]. The stronger inhibition observed against *S. aureus* compared to *E. coli* is consistent with the structural differences between Gram-positive and Gram-negative bacteria, where the thicker peptidoglycan layer of *S. aureus* allows greater interaction with released metal ions, whereas the outer lipopolysaccharide membrane of *E. coli* acts as a partial diffusion barrier [30, 53]. Figure 3b illustrates the antibacterial performance of PVA/PS-NFs, where a clear concentration-dependent increase in inhibition area was observed with increasing PS content (PS10%–PS40%). PVA/PS10-NFs showed inhibition areas of about 150–160 mm² for *E. coli* and 200–210 mm² for S. aureus, which increased to around 170–180 mm² and 250–260 mm² for PVA/PS20-NFs, and nearly *190–200* mm² and *300–310* mm² for PVA/PS30-NFs, respectively, while PVA/PS40-NFs exhibited the highest antibacterial activity with zones of roughly *230–240* mm² against *E. coli* and 380–390 mm² against *S. aureus*. This behavior is attributed to the high phenolic and flavonoid content of the plant extract, which can induce bacterial membrane permeability, protein denaturation, and oxidative stress. Phenolic compounds are known to interact with bacterial cell walls and membranes via hydrogen bonding and hydrophobic interactions, ultimately leading to leakage of intracellular constituents [54]. Accordingly, it was observed that PS-loaded NFs have the same behavior as MOF-loaded NFs, where *S. aureus* exhibited higher susceptibility than *E. coli*, further supporting the role of bacterial cell wall architecture in determining antibacterial sensitivity. The composite PVA/MOF/PS-NFs (Fig. 3c) demonstrated superior antibacterial activity compared to single-component systems, confirming a enhanced effect between MOF and PS extract. The inhibition area increased progressively with PS extract concentration. PVA/MOF5/PS40-NFs exhibited the strongest antibacterial performance of approximately 265 mm² against E. coli and 295 mm² against S. aureus, while PVA/MOF5/PS30-NFs displayed comparable behavior at around 235 mm² and 255 mm², respectively. As shown in Zheng et al. (2025), a clear dose-response relationship was established, indicating that the concentration of the active agent significantly influences its antimicrobial activity [55]. This enhanced effects can be attributed to the combined mechanisms of action, where MOF NPs facilitate sustained metal-ion release and ROS generation, while the plant extract contributes membrane-active phenolic compounds [56]. Moreover, encapsulation within the PVA-NFs matrix ensures homogeneous dispersion, prolonged contact with bacterial cells, and controlled release of active agents [57]. Such multifunctional antibacterial behavior is particularly advantageous for wound dressing applications, where broad-spectrum activity and sustained antimicrobial protection are required. Based on these results, PVA/MOF5-NFs, PVA/PS30-NFs, and PVA/MOF5/PS30-NFs exhibited the highest antibacterial activities. These formulations were therefore selected as the optimal candidates for subsequent investigations. Taken together, evaluation of fiber morphology, diameter, antioxidant capacity, and antibacterial activity indicates that the PVA/MOF5-NFs and PVA/PS30-NFs, and PVA/MOF5/PS30-NFs formulation represents the optimal composition for subsequent characterization and applications. For comparative analysis, plain PVA-NFs was also included to provide a reliable reference for assessing performance enhancements in the optimized composite NFs.

### Characterization of optimum NFs

The optimized NFs formulations, PVA-NFs, PVA/MOF5-NFs, PVA/PS30-NFs, and PVA/MOF5/PS30-NFs, were comprehensively characterized using FT-IR spectroscopy, XRD, TGA, TEM, tensile strength testing, swelling behavior, and solubility assessments. The corresponding results are presented in the following sections.

#### Fourier transform-infrared (FT-IR) spectroscopic analysis

**Fig. 4:**
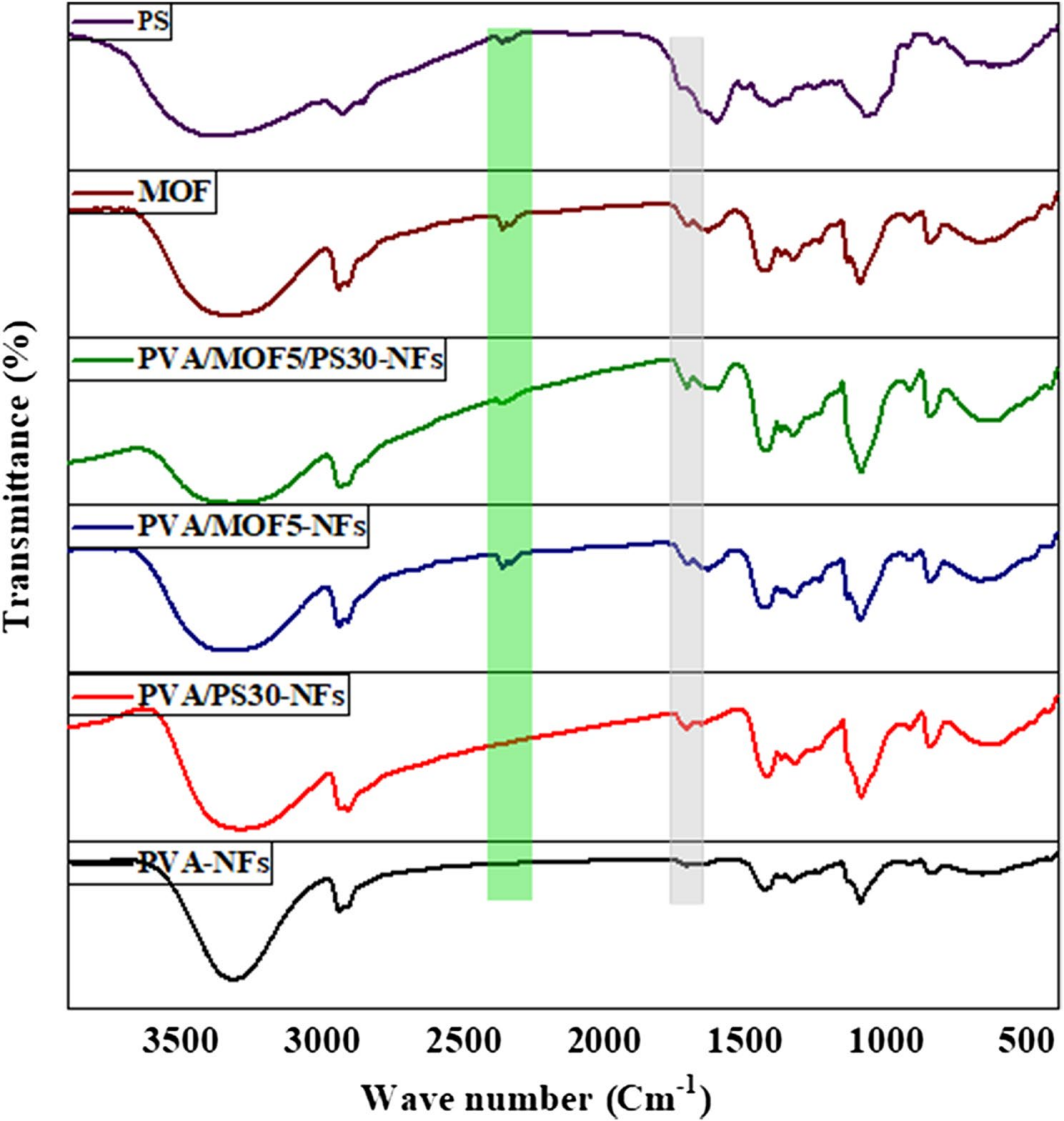
**FT-IR spectra of the prepared NFs**

FT-IR spectra of the prepared materials, including PVA-NFs, PS, MOF, PVA/MOF5-NFs, PVA/PS30-NFs, and the hybrid PVA/MOF5/PS30-NFs are presented in Fig. 4. Pure PVA-NFs displayed the typical O–H stretching band at ~ 3300–3400 cm⁻¹, C–H stretching at ~ 2900 cm⁻¹, and strong C–O and C–O–C vibrations between 1080 and 1140 cm⁻¹, consistent with reported literature on semi-crystalline PVA [58, 59]. The FT-IR spectrum of the PS extract reflects its lipid-rich composition, characterized by the predominance of fatty acid ethyl esters in the hydroethanolic extract. A broad band observed at ~ 3200–3400 cm⁻¹ is attributed to O–H stretching vibrations, which may arise from residual phenolic constituents and hydrogen-bonded hydroxyl groups. The spectrum is dominated by strong absorption bands in the 2927–2852 cm⁻¹ region, corresponding to asymmetric and symmetric stretching vibrations of –CH₂ and –CH₃ groups, confirming the presence of long aliphatic hydrocarbon chains typical of fatty acid derivatives. Additional bands around ~ 1600–1650 cm⁻¹ may be associated with minor contributions from C = C stretching or conjugated carbonyl groups. The peaks at ~ 1415–1450 cm⁻¹ and ~ 1365 cm⁻¹ correspond to C–H bending vibrations of aliphatic chains. Furthermore, the region between ~ 1000–1300 cm⁻¹ exhibits bands attributed to C–O stretching vibrations, supporting the presence of ester linkages [60–62]. The FTIR spectrum of the zinc-ascorbate MOF showed distinct COO⁻ peaks arising from the coordinated ascorbate ligand. The asymmetric and symmetric stretching of carboxylate groups appeared at ~ 1620–1650 cm⁻¹ and ~ 1380–1410 cm⁻¹, respectively, while Zn-O vibrations were observed at ~ 420–480 cm⁻¹, confirming successful metal-ligand coordination. These spectral features agree with previously reported zinc-ascorbate MOFs, which typically show characteristic shifts of the carboxylate group upon coordination with Zn²⁺ [30]. Incorporation of PS extract into PVA-NFs (PVA/PS30-NFs) resulted in evident spectral modifications. The O–H stretching band became broader and shifted to lower wavenumbers (~ 3260 cm⁻¹), indicating the formation of hydrogen bonds between PVA hydroxyl groups and phenolic constituents of PS extract. Characteristic PS extract peaks, particularly the aromatic C = C band at ~ 1604 cm⁻¹, the phenolic C–O band at ~ 1238 cm⁻¹, and the fingerprint peaks between 1000 and 1300 cm⁻¹, were also retained, though their intensities decreased due to encapsulation and partial overlap with PVA’s matrix. Such peak broadening and intensity reduction are typically associated with uniform distribution of the extract within the polymer and successful molecular interaction, consistent with previously reported plant-extract-loaded PVA- NFs [63, 64]. The appearance of MOF-specific COO⁻ vibrations at ~ 1628 cm⁻¹ and **~** 1395 cm⁻¹, along with a slight shift and broadening of the O–H band, suggests coordination interactions and hydrogen bonding between PVA and the zinc–ascorbate system. The increase in intensity of carboxylate-related peaks further confirms the presence of the crystalline MOF structure within the polymer matrix. This behavior aligns with the reported interaction between metal ions and the hydroxyl functionality of PVA, which often causes changes in both peak position and absorbance [65]. The hybrid PVA/MOF5/PS30-NFs displayed spectral contributions from all three components. The O–H stretching band exhibited significant broadening and reduction in intensity, indicating strong intermolecular interactions among PVA, phenolic compounds, and the MOF. The aromatic PS extract peaks at ~ 1604 cm⁻¹ and ~ 1518 cm⁻¹ (C = C stretching vibrations from phenolic/aromatic compounds) remained detectable alongside carboxylate vibrations from the MOF at ~ 1630 cm⁻¹ (asymmetric) and ~ 1390 cm⁻¹ (symmetric), confirming successful co-encapsulation of both components within the PVA matrix [66]. Overall, the FT-IR spectral analysis confirms the successful encapsulation of PS extract, MOF, and their hybrid combination into PVA-NFs. The observed band shifts, broadening, and changes in absorbance intensity support the presence of intermolecular hydrogen bonding, polymer-MOF coordination, and uniform distribution of bioactive compounds throughout the nanofiber matrix.

#### Thermogravimetric analysis (TGA) analysis

**Fig. 5:**
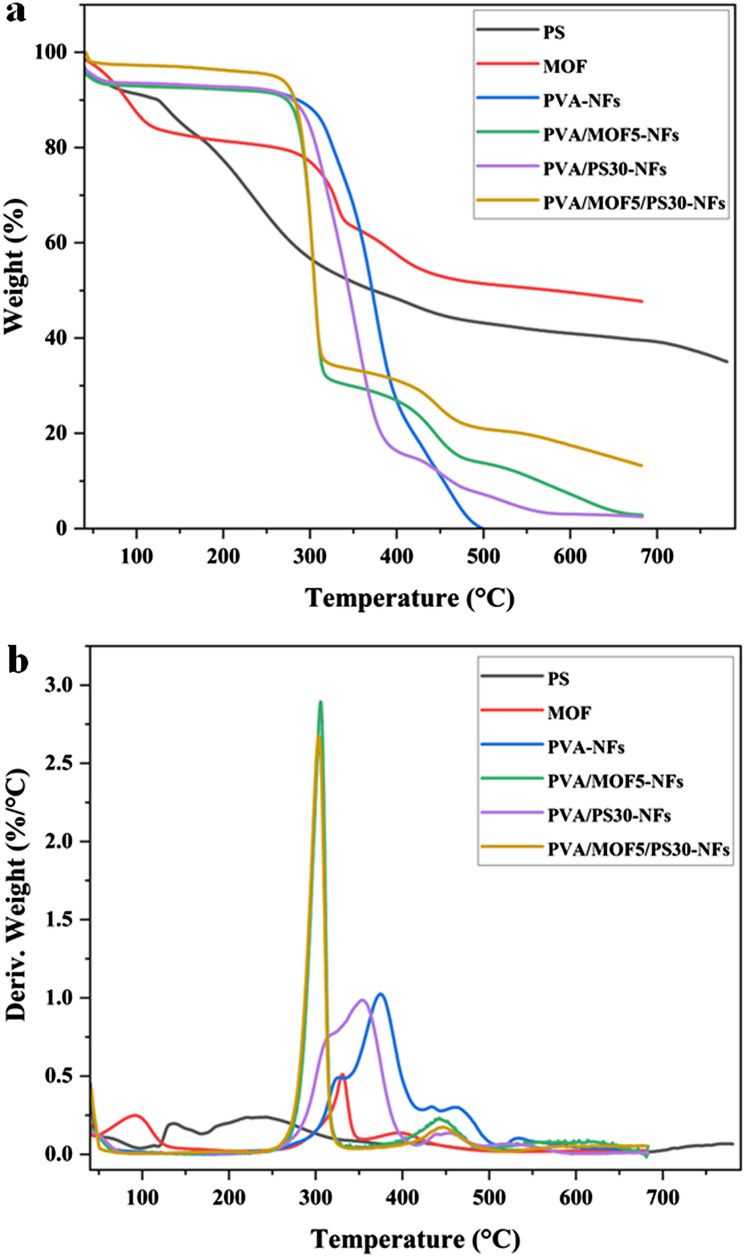
(**a**) TGA and (**b**) DTG analysis of the various prepared NFs

Thermogravimetric analysis (TGA) and derivative thermogravimetry (DTG) were conducted to systematically evaluate the thermal stability and degradation behavior of the synthesized NFs and its individual constituents, including MOF, PS extract, neat PVA-NFs, PVA/MOF5-NFs, PVA/PS30-NFs, and PVA/MOF5/PS30-NFs, as presented in Fig. 5. Pure PVA-NFs exhibited a characteristic two-step degradation profile typical of electrospun PVA-NFs systems: an initial minor weight loss of approximately 5–8% below 150 °C, attributed to the evaporation of bound and free moisture. This was followed by the major polymer backbone decomposition between 280 and 450 °C, with a prominent DTG peak around 350–380 °C representing the elimination of acetate groups and chain scission. These features align with pure PVA-NFs exhibiting three-stage degradation up to ~ 489 °C with no residue, underscoring baseline instability improved by additives [67]. Neat PS extract exhibited extensive thermal instability, characterized by nearly complete mass loss (~ 95%) within the 100–250 °C range, which underscores the thermal lability of its polyphenolic, flavonoid, and terpenoid constituents. This behavior aligns with common TGA profiles of plant extracts, which typically show 80–95% weight loss between 100 and 300 °C due to the evaporation and degradation of polyphenolics and terpenoids. For instance, TGA analysis of onion peel extract revealed multi-stage decomposition, with initial mass loss below 200 °C attributed to the early evaporation or degradation of volatile polyphenolics, flavonoids (e.g., quercetin), and terpenoids, thereby confirming their inherent thermal instability [68]. The plain MOF sample showed a more gradual degradation profile, with ~ 15–20% water loss (coordinated and adsorbed) from 50 to 200 °C. This was followed by framework decomposition spanning 250–500 °C, peaking near 370–420 °C due to organic linker breakdown, consistent with zinc-based MOF thermal signatures. This behavior aligns with the thermal signature reported for the zinc-ascorbate MOF framework in the literature, where analogous dehydration and linker combustion stages were observed [30]. Composite formulations demonstrated notable changes in thermal behavior, indicating interactions between the incorporated components. PVA/MOF5-NFs exhibited an earlier primary degradation stage, with the main DTG peak observed at approximately ~ 300 °C compared to ~ 400 °C for pure PVA-NFs. This shift suggests that the incorporation of MOF5 influences the degradation pathway of the polymer matrix. However, the composite system showed slightly higher residual weight (~ 3–5% at 600 °C) compared to nearly complete degradation of pure PVA-NFs. These changes signify MOF-induced stabilization through metal-hydroxyl coordination and restricted chain mobility. For example, Shooto et al. (2016) reports enhanced thermal stability (higher residue and decomposition points) in PVA/MOF-NFs due to successful tetracarboxylate MOFs (Sr-TBC, La-TBC, Sb-TBC) incorporation, though it focuses less on coordination mechanisms [67]. Nevertheless, PVA/PS30-NFs showed similarly improved profiles with initial degradation onset at ~ 307 °C, DTG peak maximum at ~ 420 °C, and elevated char residue (~ 10–12% at 600 °C), attributable to hydrogen bonding between extract constituents and PVA hydroxyls that hinders volatile release and enhances cross-linking during pyrolysis. For example, Encapsulation of *Mondia whitei* plant extract into PVA-NFs increased TGA stability, with MW/PVA blends showing enhanced thermal decomposition temperatures and reduced weight loss compared to pure PVA. Encapsulation of *Mondia whitei* plant extract into PVA- NFs increased TGA stability, with *Mondia whitei*/PVA blends displaying higher thermal decomposition temperatures and reduced weight loss compared to pure PVA.

This enhancement is attributed to hydrogen bonding between plant functional groups and PVA hydroxyls [69]. Most notably, the dual-loaded PVA/MOF5/PS30-NFs displayed superior thermal resistance, featuring the highest onset temperature (~ 331 °C for main degradation), significantly retarded DTG peak at ~ 465 °C with the broadest and lowest-intensity decomposition signal, and the greatest residual mass (~ 18–22% at 700 °C), confirming enhanced stabilization from combined MOF framework rigidity, extract antioxidant barriers, and multi-component interfacial bonding that collectively impede heat transfer and mass transport during thermal exposure. These progressive shifts in degradation temperatures, diminished DTG peak heights, and augmented char yields across the series align with established literature on PVA-MOF-plant extract hybrids, where such modifications reflect strong molecular-level interactions, altered degradation kinetics, and enhanced structural integrity for advanced biomedical applications.

#### X-ray Diffraction (XRD) analysis

**Fig. 6:**
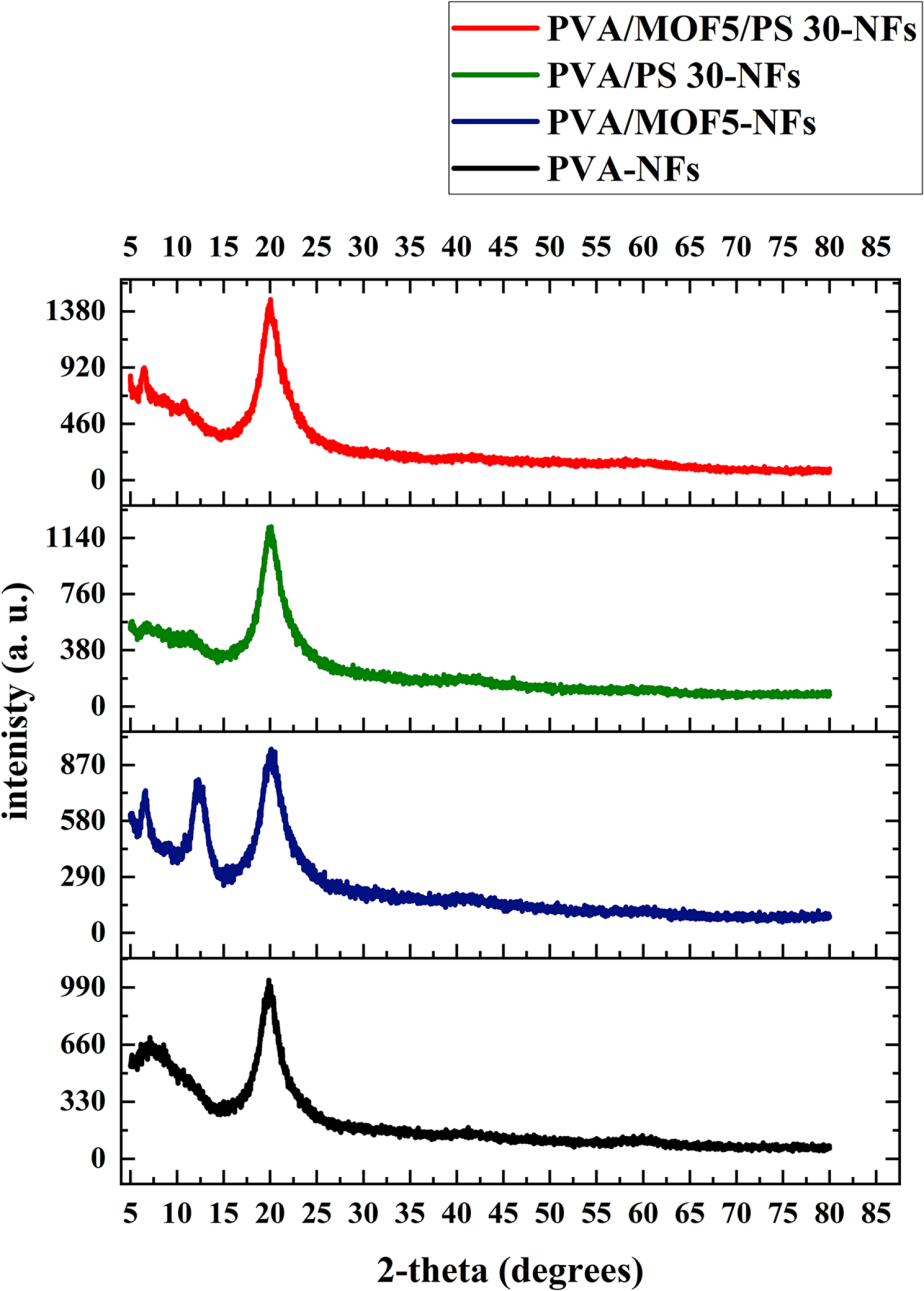
XRD analysis of the prepared NFs

XRD analysis (Fig. 6) was performed to evaluate the crystalline structure and phase composition of the as-prepared NFs samples: pristine PVA-NFs, PVA/MOF5-NFs, PVA/PS30-NFs, and PVA/MOF5/PS30-NFs. The XRD pattern of pristine PVA-NFs exhibited broad amorphous halos characteristic of their semi-crystalline nature, along with a distinct peak at 2θ ≈ 19–20°. This feature aligns with established patterns for electrospun PVA-NFs reported in the literature [69, 70]. The incorporation of MOF NPsor PS extract resulted in noticeable changes in the diffraction patterns; PVA/MOF5-NFs and PVA/PS30-NFs both showed a discernible enhancement of crystallinity, as evidenced by the sharper and more intense peaks near 2θ ≈ 19–23°, likely due to interactions between PVA chains and the added functional phases. Additionally, PVA/MOF5-NFs exhibited three characteristic peaks at 2θ ≈ 5°, 15°, and 20°, whereas PVA/PS30-NFs displayed a minor peak at 2θ ≈ 5°. These observations confirm the successful encapsulation of MOF within the prepared nanofibers and are consistent with previously reported XRD patterns of MOF by Haikal et al. [30]. These findings agree with previous reports on MOF-embedded polymeric NFs, where shifts and amplified peaks signify effective loading and physical interactions with the matrix [71, 72]. In summary, XRD analysis confirms the successful structural incorporation of MOF and PS extract into the PVA nanofiber matrix, as evidenced by peak shifts, intensity changes, and the preservation of functional crystalline motifs after processing.

#### Transmission Electron Microscope (TEM analysis)

**Fig. 7:**
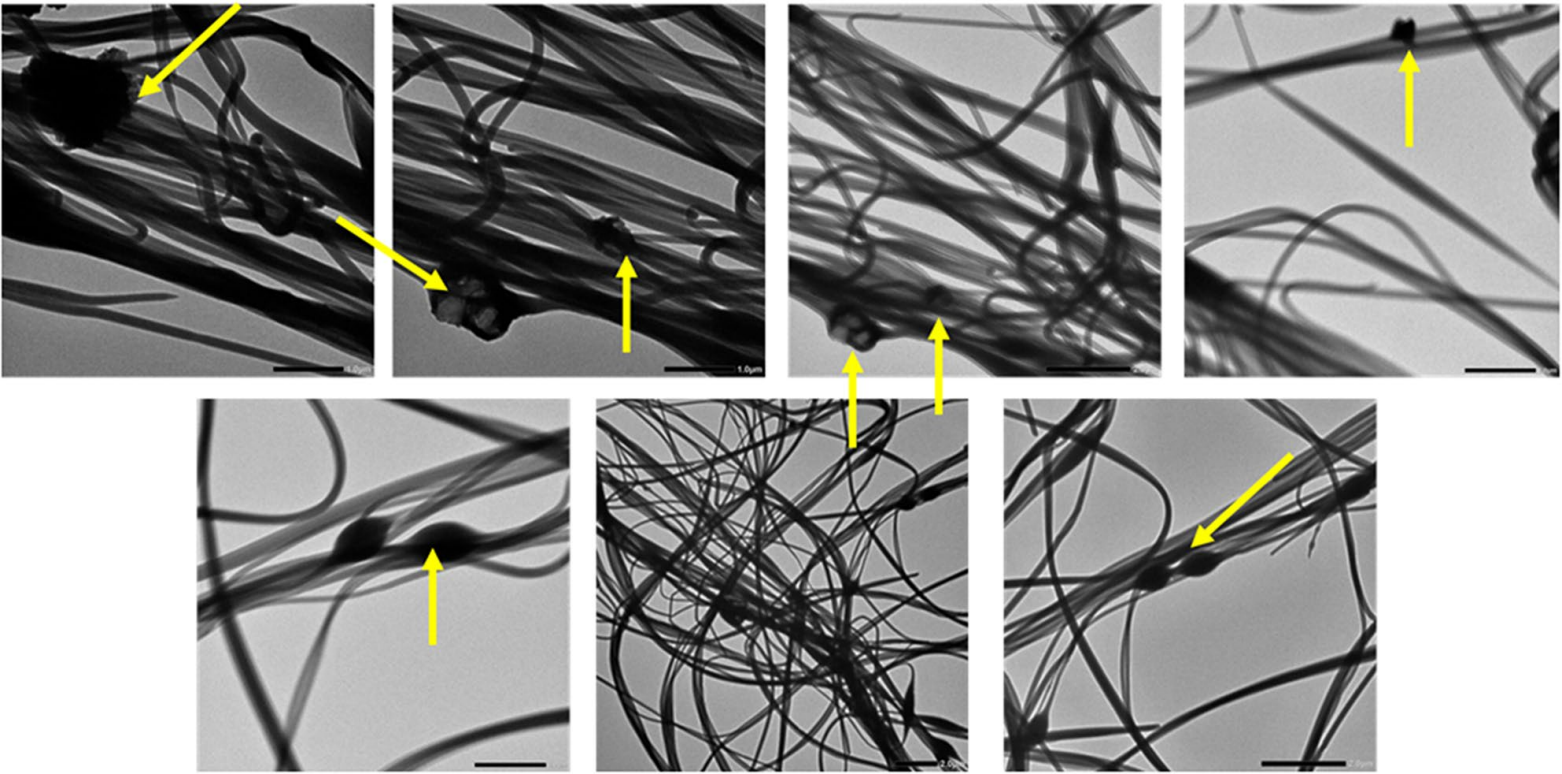
TEM analysis for the optimum prepared PVA/MOF5/PS30-NFs, showing the immobilization of the MOF NPs in the NFs from different areas

TEM (Fig. 7) imaging of the optimum hybrid NFs (PVA/MOF5/PS30-NFs) reveals a well-defined fibrous structure with uniform diameter and distinct encapsulated particles embedded within the NF matrix, as indicated by the highlighted regions. These embedded domains represent successful immobilization of MOF and PS components throughout the PVA-NFs, which is evidenced by the presence of darker contrast features corresponding to the denser, encapsulated phases. The morphology, showing discrete and continuous distribution of loaded particles across different fields, confirms effective encapsulation consistent with previous reports on MOF/polymer and plant extract/polymer NF composites [73]. Such TEM observations substantiate both the physical integration and dispersity of active components within the polymer framework, reflecting the homogeneous mixing and structural preservation required for advanced biomedical and environmental applications.

#### Tensile strength

The mechanical properties of the prepared NFs, assessed for the optimum NFs formulas using a tensile stage system, and the results are summarized in Table 4, displaying NFs diameter, tensile strength and elongation percentage values. Data revealed that tensile strength increased upon immobilization of PS and MOF into PVA-NFs, reaching the highest value of 14.95 MPa with 136% elongation for the PVA/MOF5/PS30-NF formulation in contrast to PVA-NFs with values of 2.63 MPa and 13.8% for tensile strength and elongation percent, respectively. This enhancement aligns with polymer composite systems where nanofillers reinforce the matrix; specifically, electrospun NFs based on plant extract bioactive materials exhibit improved tensile strength upon plant extract immobilization [74]. For example, literately reported PVA-CNF-MOF composite films show both tensile strength and Young’s modulus increasing with CNF content, peaking at 5% incorporation due to inter- and intramolecular hydrogen bonding, uniform matrix dispersion, and high PVA compatibility [75]. The underlying mechanism supports the current findings, where optimal plant extract and MOF loading strengthens the PVA nanofiber framework through compatible interfacial interactions, whereas excessive concentrations risk aggregation and diminished performance beyond the optimal threshold.

**Table 4:** Tensile strength data of the prepared nanofibers

NFs-formulations	NFs-Diameter (mm)	Elongation percent (%)	Tensile strength (MPa)
PVA-NFs	0.06	13.8	2.63
PVA/MOF5-NFs	0.055	117	1.88
PVA/PS30-NFs	0.11	61.65	1.12
PVA/MOF5/PS30-NFs	0.055	136	14.95

#### Swelling and solubility

**Fig. 8:**
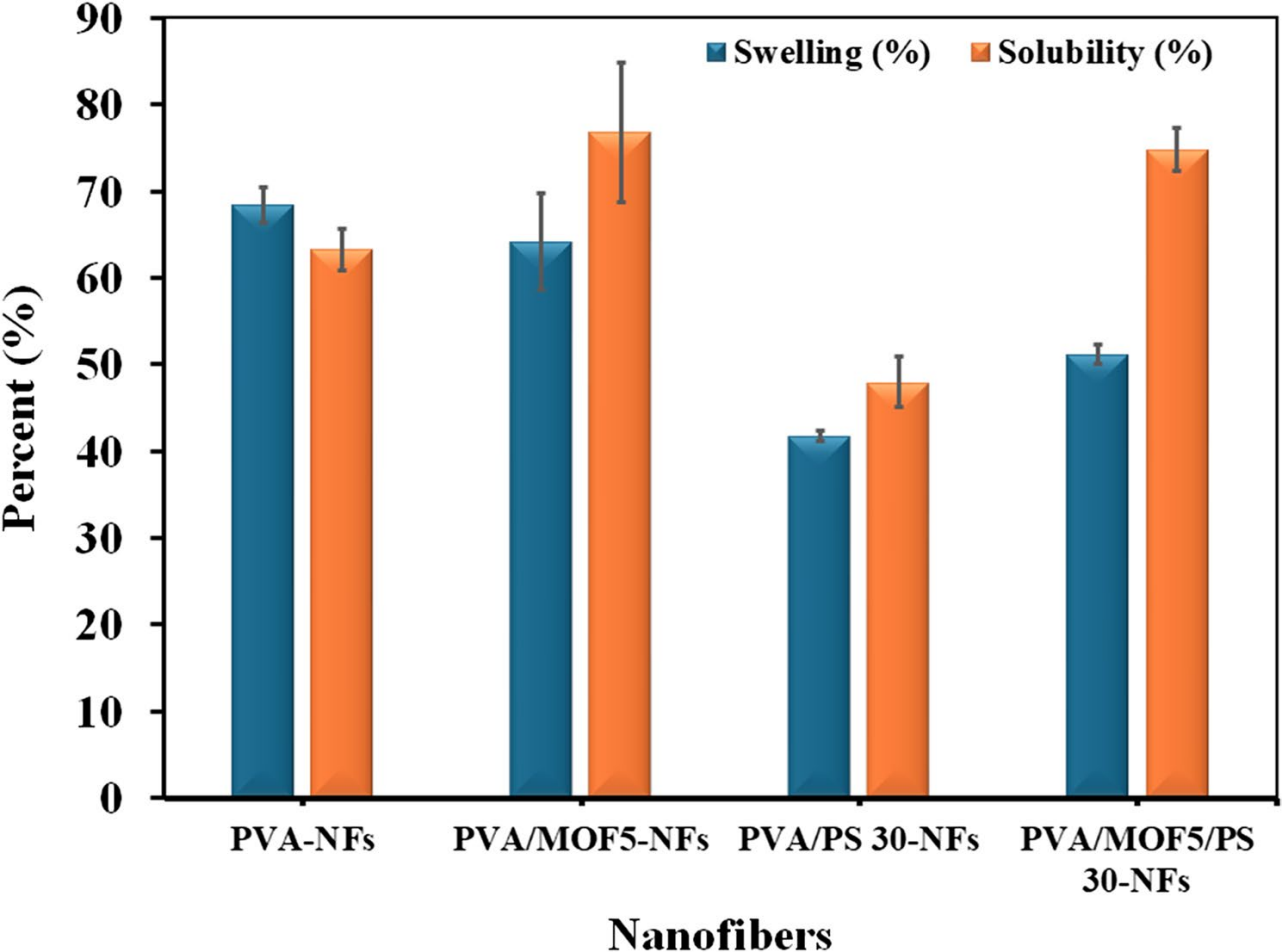
Swelling and solubility of the optimum prepared NFs

The swelling and solubility properties (Fig. 8) of the synthesized NFs were analyzed to assess their aqueous stability and matrix interaction effects. Pristine PVA-NFs exhibited a high swelling ratio (approximately 68%) and solubility (~ 63%), reflecting the inherent hydrophilic and water-soluble nature of the PVA polymer, as widely reported in electrospun fiber systems. Incorporation of MOF NPs (PVA/MOF5-NFs) resulted in comparable swelling (~ 64%) but slightly increased solubility (~ 76%), which may be attributed to the presence of MOF domains that enhance water uptake and dissolution by disrupting the polymer network. Loading with PS extract (PVA/PS30-NFs) led to marked reductions in both swelling (~ 44%) and solubility (~ 52%) versus pure PVA-NFs. This decrease is likely attributed to the lipophilic nature of the extract, as previously demonstrated by GC-MS analysis, which effectively increases the overall hydrophobicity of the nanofiber matrix. The ternary hybrid PVA/MOF5/PS30-NFs also demonstrated reduced swelling (~ 53%) compared to pure PVA-NFs, but high solubility (~ 73%), suggesting enhanced effects where MOF increases dissolution, while PS extract crosslinks the matrix and moderates’ expansion. These findings support the hypothesis that both MOF and plant-based additives can tune the water-responsive behavior of PVA-NFs via complex physical and chemical interactions, aligning with studies showing that MOF incorporation into PVA increases surface area and alters physicochemical properties such as swelling and water uptake due to enhanced polymer-MOF interactions [76]. This behavior also aligns with literature on plant extract-loaded electrospun fibers, where the introduction of bioactive compounds influences hydrophilicity, swelling, and solubility through competitive water-polymer and additive-polymer interactions [74].

#### Total phenolic content (TPC)

The total phenolic content (TPC) results (Table 5) indicate substantial retention of PS extract phenolics in PVA-NFs, particularly upon hybrid MOF/PS incorporation. Pure PS extract displayed 29.80 mg GAE/g TPC, MOF showed 66.25 mg GAE/g from precursor phenolics, while pristine PVA-NFs had negligible 0.06 mg GAE/g. PVA/MOF5-NFs retained 3.53 mg GAE/g, PVA/PS30-NFs achieved 0.66 mg GAE/g, and the ternary PVA/MOF5/PS30-NFs reached 1.41 mg GAE/g, demonstrating effective co-immobilization despite processing losses.

#### IC_50_/Antioxidant activity

The antioxidant activity of the prepared NFs was evaluated by determining their IC_50_ values (the concentration required to inhibit 50% of free radicals) at both 2 h and 24 h. PS extract demonstrated the lowest IC_50_ values (3.57 and 3.50 mg/3mL for 2 and 24 h, respectively), reflecting potent radical-scavenging capacity as expected for plant-derived antioxidants. Pristine PVA-NFs exhibited markedly higher IC_50_ values (69.94 and 44.95 mg/3mL, respectively), indicating weak antioxidant performance due to the lack of bioactive components. Incorporation of MOF (PVA/MOF5-NFs) substantially reduced the IC_50_ values (12.82 and 7.09 mg/3mL, respectively), confirming the role of zinc ascorbate-based MOF in enhancing antioxidant activity. Loading PS extract into PVA (PVA/PS30-NFs) yielded IC_50_ values (20.75 and 20.47 mg/3mL, respectively), which were lower than pure PVA but higher than MOF composites, showing moderate antioxidant enhancement likely driven by polyphenolic content. The ternary hybrid (PVA/MOF5/PE30-NFs) exhibited enhanced improvement, with IC_50_ values (15.17 and 11.11 mg/3mL, respectively), outperforming single-loaded PVA/PS-NFs and confirming the increased effectiveness of integrating both MOF and plant-derived antioxidants into the matrix. Overall, these results demonstrate that NF antioxidant capacity is greatly influenced by the type and combination of loaded active agents, MOF and PS extract significantly improved IC_50_ compared to pure PVA, which was found to be in agreement with literature findings on MOF, and plant-loaded PVA-NFs.

**Table 5:** IC50 activity of the optimum prepared nanofibers

Sample	TPC (mg/g GAE)	IC_50_ at 2 h. (mg/3 ml)	IC_50_ at 24 h. (mg/3 ml)
PS	29.80	3.57	3.50
MOF	66.25	-	-
**PVA-NFs**	**00.06**	**69.94**	**44.95**
**PVA/MOF5-NFs**	**03.53**	**12.82**	**7.09**
**PVA/PS30-NFs**	**00.66**	**20.75**	**20.47**
**PVA/MOF5/PS30-NFs**	**01.41**	**15.17**	**11.11**

### Release profiles and kinetics

**Fig. 9:**
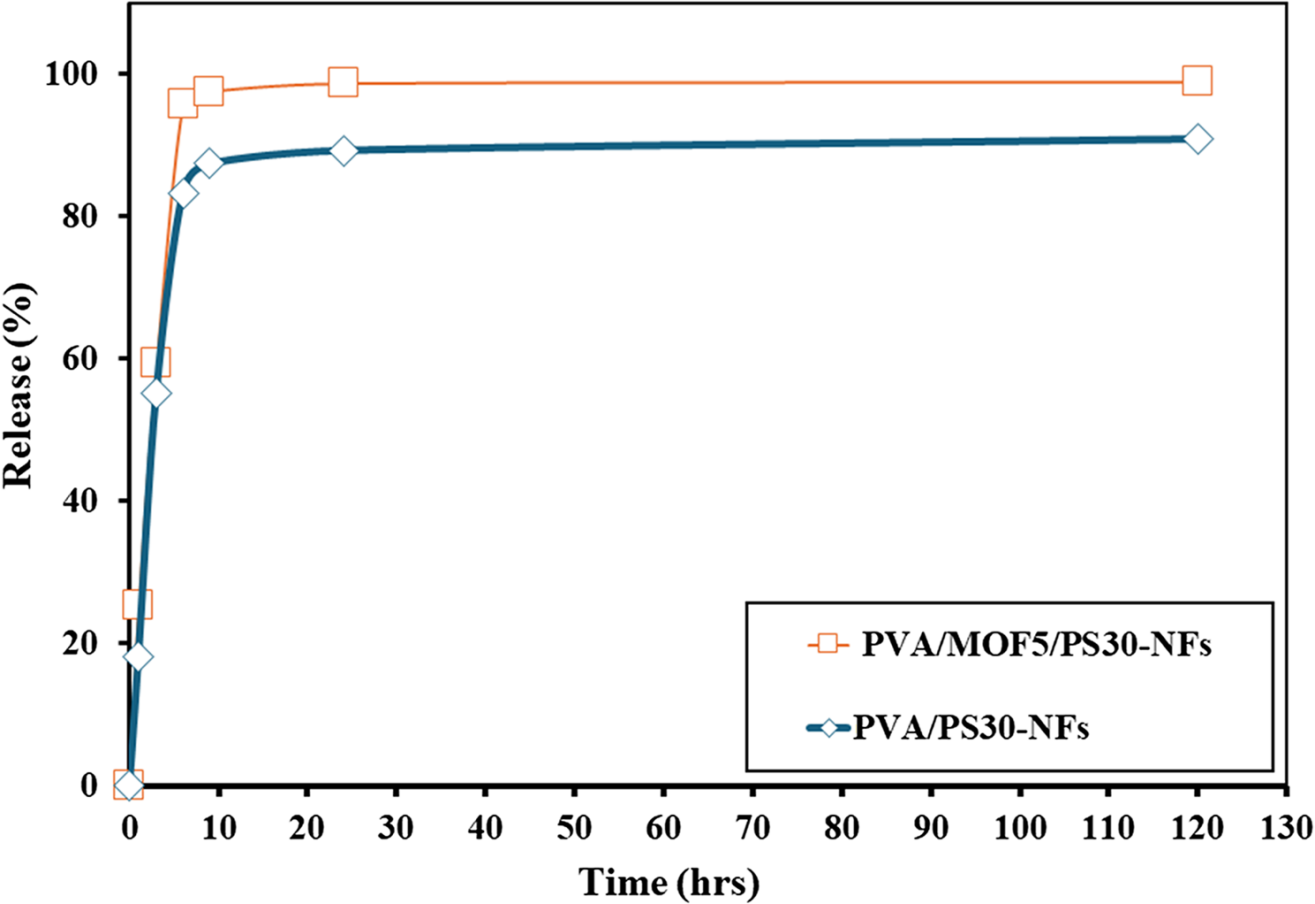
In-vivo wound healing evaluation of the different prepared NFs

**Fig. 10:**
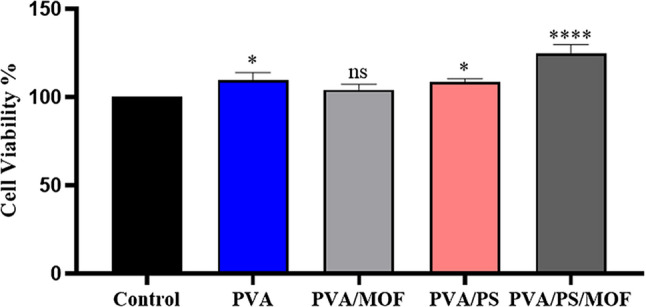
Release profile of (**a**) PVA/MOF-NFs & PVA/PS-NFs, (**b**) PS in PVA/MOF/PS-NFs & MOF in PVA/MOF/PS-NFs

The in vitro release profiles of the PS extract from the PVA-based nanofiber systems (Fig. 9) exhibited distinct biphasic patterns over observation periods ranging from 10 to 120 h. For the PVA/PS30-NFs, an initial burst release of approximately 18% was observed within the first hour, followed by a sustained release phase that reached ~ 90% after 9 h. This behavior is primarily attributed to the rapid disruption of extract molecules from the fiber surface and subsequent Fickian diffusion through the swollen PVA matrix. A similar burst release mechanism was previously documented for *Calendula officinalis* extract-loaded PLLA/HPC nanofibers, where rapid surface desorption led to a cumulative release plateau of 0.89 ± 0.02 within 540 min (9 h) in PBS (pH 7.4, 37 °C) [77]. In contrast, the hybrid PVA/MOF5/PS30-NFs composite (Fig. 10) displayed a significantly modified release trajectory. While the PS extract showed a rapid initial phase exceeding 90% within the first 6 h, it was followed by a substantially more prolonged release phase that achieved 100% cumulative delivery at approximately 120 h. This extended-release duration suggests that the incorporation of MOF creates a more complex diffusion path or provides additional binding sites, effectively regulating the out-flux of the bioactive components and providing a superior long-term delivery profile compared to the single-component system.

The release kinetics of the PS extract from the PVA-based nanofiber systems were systematically analyzed using various mathematical models, with the resulting correlation coefficients (R²) summarized in Table 6. For the single-component PVA/PS30-NFs, the data showed exceptional agreement with both the Higuchi (R² = 0.99) and Korsmeyer-Peppas (R² = 0.99) models. These high values confirm that the release of small molecules from the single-component fibers is primarily governed by Fickian diffusion, where the bioactive molecules migrate through the swollen polymer matrix along a concentration gradient. In contrast, the hybrid PVA/MOF5/PS30 composite nanofibers exhibited a significant shift in release behavior. The Hixson-Crowell model (R² = 0.83) provided a superior description of the kinetics compared to the Higuchi model, suggesting the simultaneous involvement of diffusion and polymer matrix erosion. This transition indicates that the incorporation of MOF into the composite structure introduces a more complex interplay of mechanisms, where release is controlled not only by diffusion but also by polymer relaxation and the structural degradation of the nanofibrous framework. These findings highlight the role of the MOF component in transforming the delivery system from a simple monolithic diffusion matrix into a sophisticated, erosion-controlled hybrid vehicle [78–80].

**Table 6:** Kinetic model parameters for drug release from prepared PVA-NFs samples

Model	PVA/PS30-NFs	PVA/MOF5/PS30-NFs
Zero order kinetics	0.96	0.43
1st order kinetics	0.83	0.44
Higuchi	0.99	0.64
Hixon Crowell	0.83	0.83
Kors-peppas	0.99	0.81

### Wound healing assay

#### In-vitro cell viability and wound healing cell proliferation assays

**Fig. 11:**
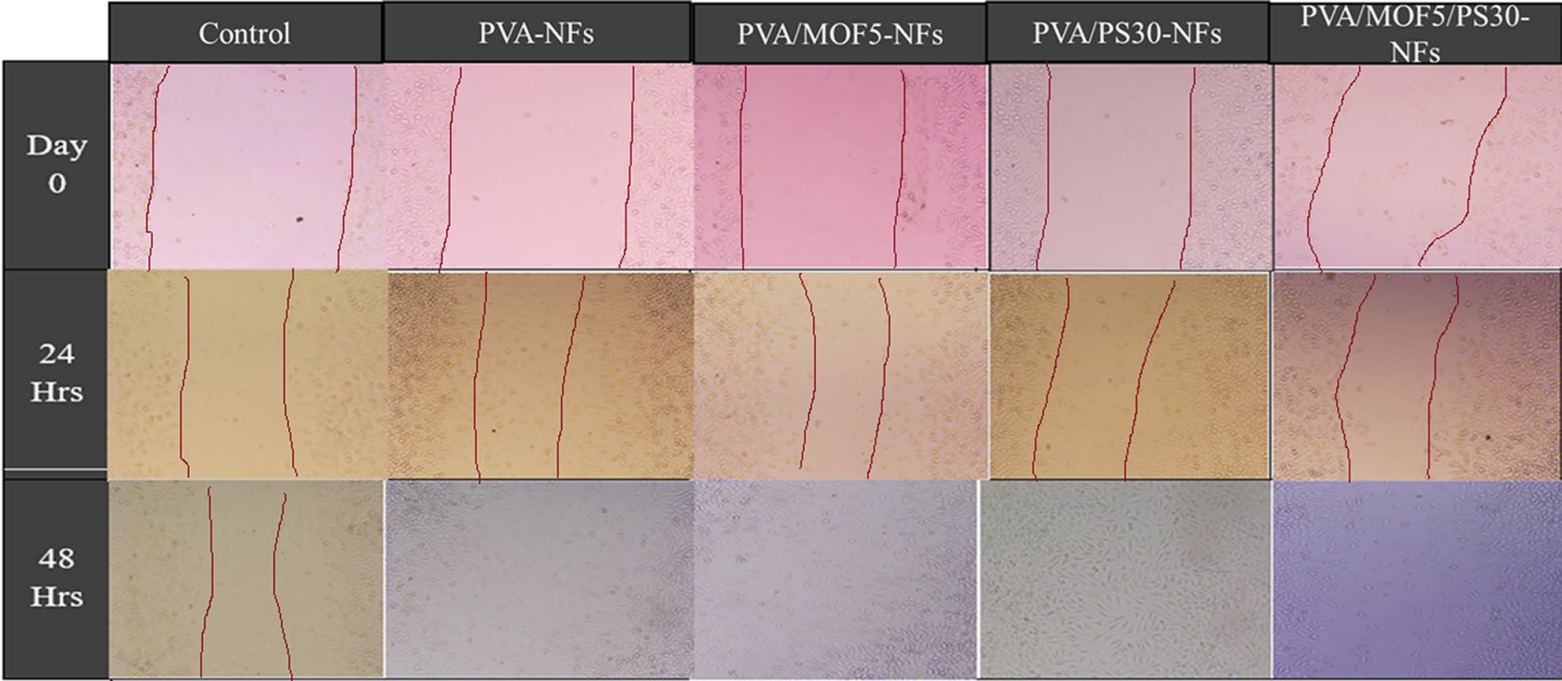
In-vitro cell viability assessment of the prepared NFs composites

The MTT assay results demonstrate high biocompatibility of the tested nanofibrous scaffolds with fibroblast cells, Fig. 11. The control and PVA groups exhibited approximately 100% viability, while PVA/MOF5-NFs and PVA/PS30-NFs maintained viabilities around 100% (non-significant differences, ns), and PVA/MOF5/PS30-NFs showed the highest viability exceeding 100% with highly significant elevation (**p < 0.0001). These findings, supported by statistical markers (*p < 0.05 where applicable), confirm no cytotoxic effects across treatments, aligning with literature on PVA-based composites in fibroblast viability assays [81].

#### In-vitro assay (scratch assay) for the various prepared composites

**Fig. 12:**
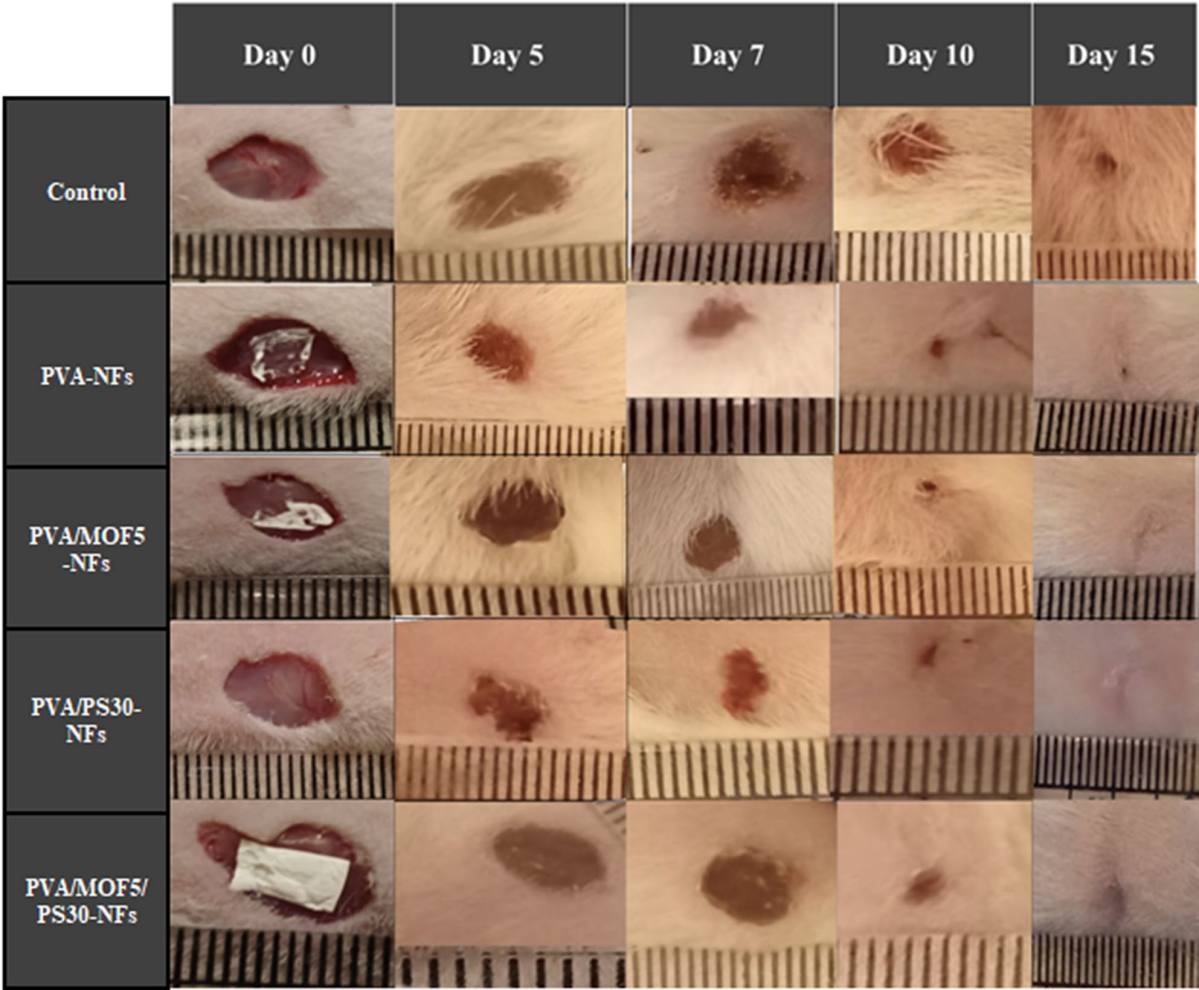
In vivo wound closure percentages of the prepared NFs

The in-vitro scratch assay (magnification 400×) on L929 fibroblast cells illustrates the wound closure progression across control and nanofibrous scaffolds (PVA-NFs, PVA/MOF5-NFs, PVA/PS30-NFs, PVA/MOF5/PS30-NFs) at 0, 24, and 48 h, as depicted in Fig. 12. At t = 0, uniform open scratches appear across all groups with pink cell monolayers; by 24 h, treated groups exhibit noticeable edge contraction and fibroblast migration into the wound bed, outperforming the static control; and at 48 h, substantial closure occurs in PVA-NFs, PVA/MOF5-NFs, PVA/PS30-NFs, and PVA/MOF5/PS30-NFs, with denser cellular infiltration relative to control. These observations indicate significantly enhanced cell migration and proliferation in nanomaterial-treated groups, promoting faster wound healing compared to untreated controls, consistent with prior reports on PVA-based electrospun NFs accelerating fibroblast-mediated regeneration in scratch models. PVA-NFs, often modified with MOFs or PS extract, provide biomimetic topography that stimulates focal adhesion and cytoskeletal dynamics in fibroblasts, thereby boosting migration rates as quantified in similar L929 assays. The observed temporal progression aligns with literature demonstrating PVA composites’ role in upregulating proliferation markers and extracellular matrix deposition, essential for regenerative applications like wound dressings [82, 83]. These biocompatible scaffolds (corroborated by prior MTT data) hold promise for advanced drug delivery in tissue engineering, warranting in-vivo validation.

#### In-vivo wound healing assay of the prepared NFs

**Fig. 13:**
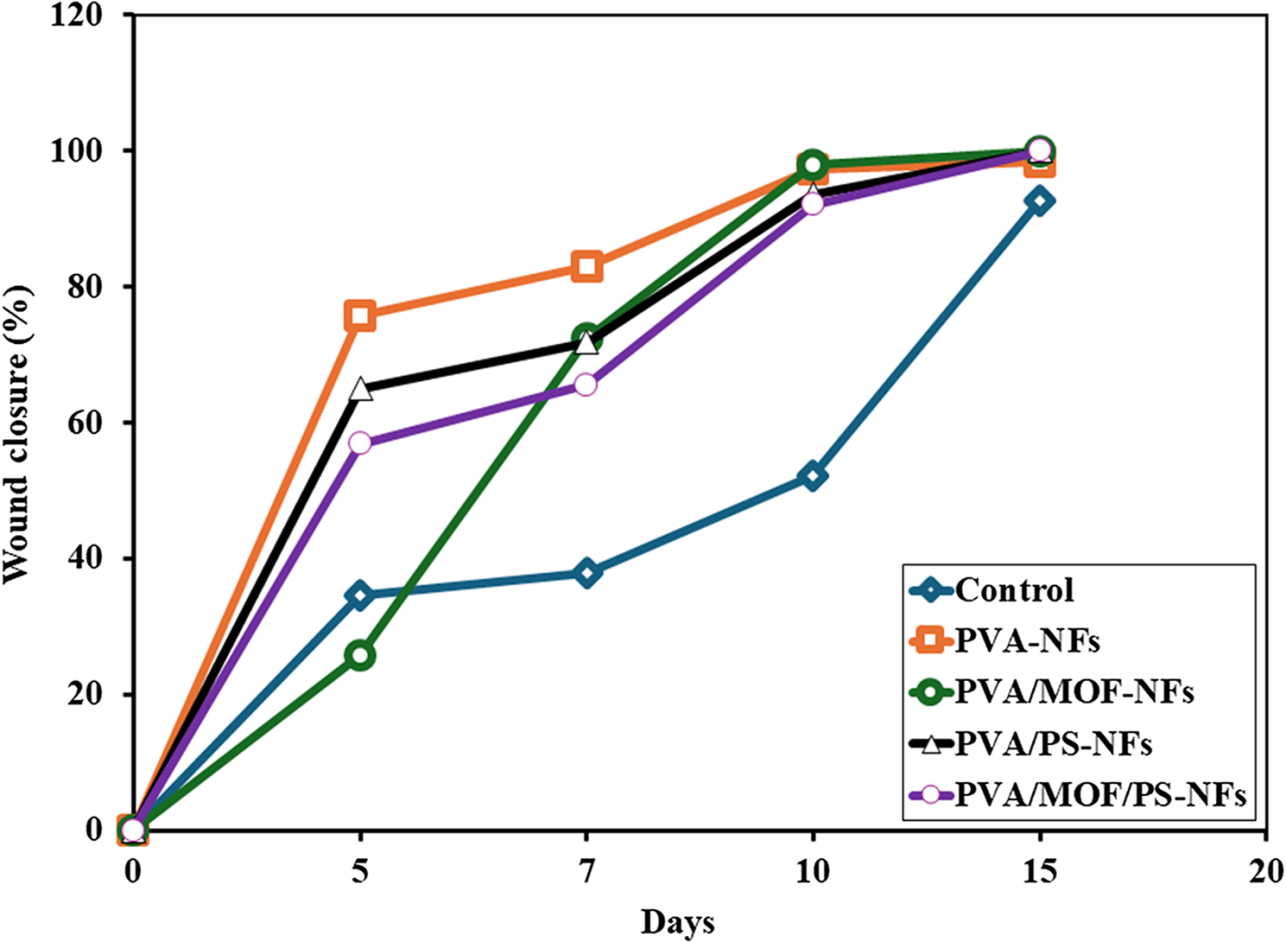
In-vitro wound closure percentages of the prepared NFs

Complementing the in-vitro results, in-vivo wound healing studies in a rat excision model revealed superior granulation tissue formation and absence of infection across all treated groups throughout the 15-day observation period, as visualized in representative photographs (Fig. 9). The untreated control exhibited delayed closure with persistent open wounds at days 5–10 and only partial healing (~ 80% closure) by day 15, contrasting sharply with treated groups where PVA-NFs, PVA/MOF5-NFs, PVA/PS30-NFs, and PVA/MOF5/PS30-NFs showed accelerated contraction and re-epithelialization, particularly evident from day 5 onward. Quantitative analysis of wound closure percentages was determined via Image J software and presented in Fig. 13. This data reveals that all treated groups (PVA-NFs: orange; PVA/MOF5-NFs: green; PVA/PS30-NFs: purple; PVA/MOF5/PS30-NFs: blue) achieved over 100% closure by day 15, surpassing the control (black, ~ 80%). Additionally, PVA/MOF5/PS30-NFs exhibited the steepest trajectory and earliest acceleration post-day 5. Photographic evidence confirms this: open wounds at day 0 contract markedly in treated groups by day 5 (reduced scab size, emerging hair follicles), progressing to near-complete re-epithelialization by day 15, unlike the persistent open control wound. These findings align with electrospun PVA-NFs’ role in mimicking extracellular matrix to promote fibroblast infiltration, angiogenesis, and collagen deposition, as previously reported in rat models [84]. The superior performance of PVA/MOF5/ PS30-NFs composites likely stems from enhanced antimicrobial and bioactive ion release, reducing inflammation and ROS while enhancing vascularization, consistent with studies on PVA-MOF dressings achieving > 90% closure by day 15 [70]. Overall, integration with prior in vitro biocompatibility (MTT), migration data (scratch assay), and the in-vivo studies position PVA/MOF5/ PS30-NFs as promising bioactive scaffolds for chronic wound management, warranting clinical evaluation.

#### Histological evaluation of wound healing

**Fig. 14:**
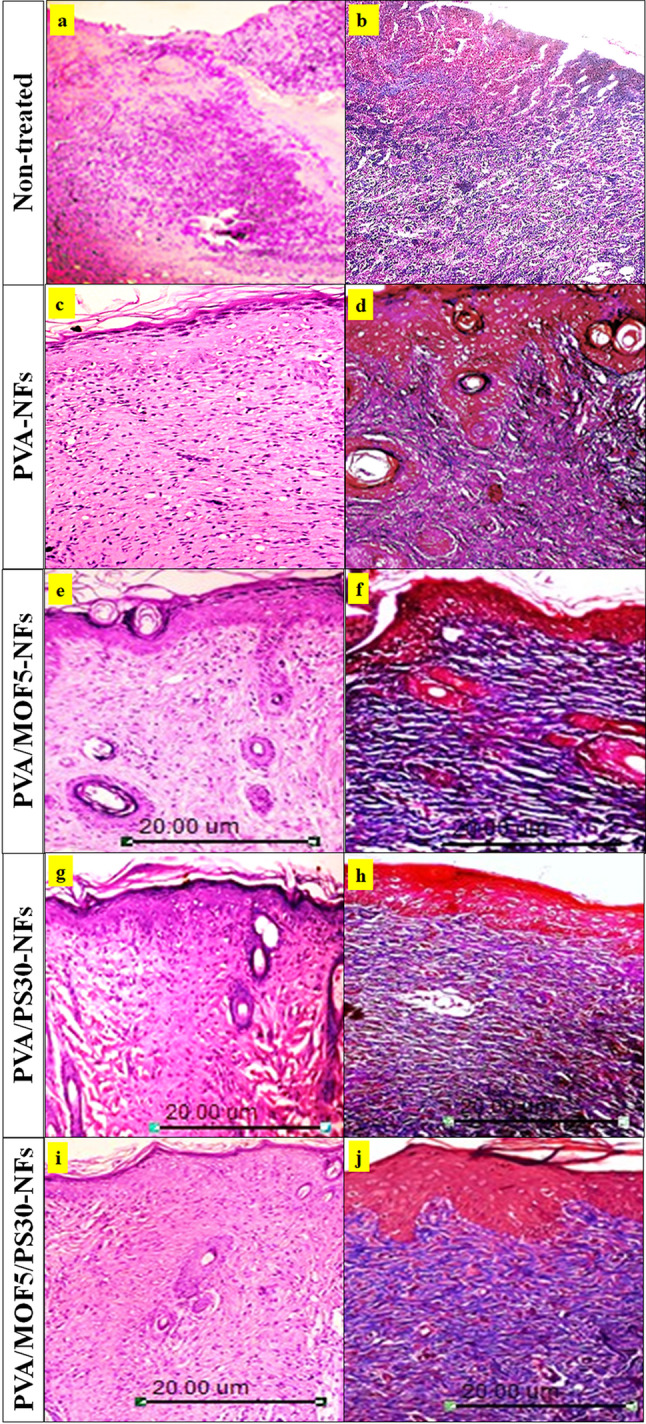
Histological evaluation of wound healing using Hematoxylin & Eosin and Masson’s trichrome stains

Histological analysis of H&E- and Masson’s Trichrome-stained skin sections from excisional wound sites revealed progressive tissue regeneration across the treatment groups, with notable improvements in re-epithelialization, inflammation resolution, granulation tissue maturation, neo-angiogenesis, and collagen deposition compared to the non-treated controls. In the normal control group, skin sections exhibited intact epidermal architecture consisting of stratified squamous epithelium with four well-defined keratinocyte layers. The basal cell layer and a thin stratum granulosum were clearly identifiable, covered by a delicate eosinophilic keratin layer. The underlying dermis consisted of dense fibrous connective tissue interspersed with subcutaneous adipose tissue and few blood capillaries, with no evidence of inflammatory responses (score 0, Table 7). In contrast, skin sections from the wound model group displayed necrotic tissue at the wound margins, with intense infiltration of inflammatory cells, mainly polymorphonuclear leukocytes, and pronounced interstitial edema (score 4). A limited number of fibroblasts (score 1), minimal neo-angiogenesis (score 2), and scant collagen fibers (score 1, Table 7) were observed near the excision site. The PVA-NFs treated group exhibited well-organized granulation tissue and pronounced epidermal regeneration at the wound edges (score 4). The dermis displayed mild inflammatory infiltration (score 1), mature granulation tissue at the wound base (score 3), mild neo-angiogenesis (score 1), and abundant new collagen fibers (score 3, Table 7). In animals treated with PVA/MOF5-NFs, complete epidermal regeneration was observed, characterized by a continuous epithelial layer covered by a thin keratin coat (score 4). Only a few infiltrating macrophages were noted (score 1). The dermis showed mild neo-angiogenesis (score 1) and well-organized granulation tissue (score 2). Masson’s Trichrome staining revealed well-aligned and mature collagen fibers within the dermal matrix (score 4, Table 7). Similarly, the PVA/PS30-NF group exhibited complete epidermal regeneration covered by a thin eosinophilic keratin layer (score 4). Mild infiltration of inflammatory cells, primarily neutrophils and macrophages (score 2), was observed, accompanied by neo-angiogenesis (score 1), extensive granulation tissue formation (score 3), and moderately organized collagen fibers (score 3, Table 7). Animals treated with the combined PVA/MOF5/PS30-NFs formulation also showed complete epidermal regeneration, with the epidermis covered by a thin keratinized layer (score 4). Minimal inflammatory infiltration, mainly macrophages (score 1), was evident. The dermal layer exhibited mild neo-angiogenesis (score 1) and well-developed granulation tissue (score 2). Collagen fibers were moderately organized as confirmed by Masson’s Trichrome staining (score 3, Table 7). In summary, *non-treated animals* (Fig. 14a, b) exhibited necrotic tissue at wound edges, accompanied by significant inflammatory cell infiltration and sparse fibroblasts and new blood vessels. *PVA-NF*-treated animals (Fig. 14c, d) demonstrated well-organized granulation tissue with prominent epidermal regeneration at wound margins, mature granulation tissue at the wound base, mild neo-angiogenesis, and mature collagen deposition as revealed by Masson’s Trichrome staining (Fig. 14d). Treatment with *PVA/MOF5-NFs* (Fig. 14e, f), *PVA/PS30-NFs* (Fig. 14g, h), and *PVA/MOF5/PS30-NFs* (Fig. 14i, j) resulted in complete epidermal regeneration covered by a thin eosinophilic keratin layer and minimal inflammatory cell infiltration. Notably, PVA/MOF5-NF-treated wounds displayed regularly arranged mature collagen fibers in the dermal layer (Fig. 14f), while *PVA/PS30-NF* and *PVA/MOF5/PS30-NF* groups showed moderately organized collagen fibers (Fig. 14h, j).

**Table 7:** Histological scoring of wound healing

Groups	Histological scale of wound healing				
	Epithelization	PMNL	Fibroblasts	New vessels	Collagen
**Normal control**	Normal histological structure of skin layers without significant pathological changes				
**Non-treated**	**0**	**4**	**1**	**2**	**1**
PVA-NFs	**4**	**1**	**3**	**1**	**3**
PVA/MOF-NFs	**4**	**1**	**3**	**1**	**3**
PVA/PS-NFs	**4**	**2**	**3**	**1**	**3**
PVA/MOF/PS-NFs	**4**	**1**	**2**	**1**	**3**

## Conclusion

A unique composition of wound healing NFs mats composed of systematically optimized MOF and PS extract concentrations incorporated within PVA NFs was carried out for the first time. This novel PVA/MOF5%/PS30% formulation exhibited superior wound healing capabilities. These NFs exhibited uniform, bead-free morphology with 167.6 nm average diameter with enhanced tensile strength of 14.95 MPa with 136% elongation. Enhanced antioxidants and antibacterial performance were also observed. This optimal NFs scaffold demonstrated substantial total phenolic content retention (1.41 mg GAE/g), low IC_50_ values (15.17 mg/mL at 2 h, 11.11 mg/mL at 24 h, respectively), and robust antibacterial inhibition zones (~ 250 mm²) against *E. coli* and *S. aureus*, outperforming single-component controls through complementary bioactive mechanisms. Comprehensive physicochemical characterization, including FTIR spectroscopy, TGA, XRD, and swelling/solubility analyses, were performed, and confirmed successful co-immobilization, thermal stability, and Hixson-Crowell release kinetics (R² = 0.83) in the hybrid system. In-vitro bio-compatibility assessments showed excellent cytocompatibility. L929 fibroblast viability exceeded 100% (***p < 0.0001). Scratch assays demonstrated accelerated wound closure with substantial migration/proliferation by 48 h versus controls. These findings translated effectively in-vivo, where rat excisional wound models demonstrated > 100% closure by day 15 for all NFs treatments, led by PVA/MOF5/PS30, contrasted against ~ 80% control healing, which was evidenced by rapid granulation, re-epithelialization, and infection-free progression. Moreover, histopathological validation further corroborated these outcomes, revealing superior collagen deposition, neovascularization, and reduced inflammation in MOF/PS composites. Collectively, this multifaceted evaluation establishes PVA/MOF5/PS30 NFs as a bioactive, mechanically robust platform enhanced plant-derived antioxidants, antimicrobial MOF activity, and biomimetic topography for advanced wound dressings. These translationally promising scaffolds bridge critical gaps in chronic wound management, warranting scale-up, clinical trials, and broader therapeutic exploration in tissue regeneration and drug delivery applications. Future studies should include detailed investigation of cell–material interaction mechanisms and confirmation of the observed metabolic stimulation using complementary assays such as live/dead staining and fluorescence imaging. In addition, extending this work to alternative biodegradable polymeric nanofiber systems, such as PCL or PLGA incorporated with MOF and PS extract, may further expand the applicability and translational potential of the developed strategy for advanced wound healing applications.

## Supplementary Information

Below is the link to the electronic supplementary material.

Supplementary Material 1

## Data Availability

No datasets were generated or analysed during the current study.
