# Supplementary material for: Combined bioactive PVA nanofibrous scaffolds loaded with Zn ascorbate MOFs and Petroselinum crispum (Parsley) extract for enhanced in-vitro and in-vivo wound healing applications
Source: J Biol Eng. 2026 Aug 17;20:127. doi: 10.1186/s13036-026-00736-8 (PMC13479814; doi:10.1186/s13036-026-00736-8)
Supplement: Supplementary file 1 [file 13036_2026_736_MOESM1_ESM.docx]

**Figure S1: SEM analysis of PVA/MOF1.25-NFs concentration (A) shows NFs at a magnification of 2 KX, (B) 10 KX magnification, and (C) shows the histogram of the plotted diameter data of image J software.**

**Figure S2: SEM analysis of PVA/MOF2.5-NFs concentration (A) shows NFs at a magnification of 2 KX, (B) 10 KX magnification, and (C) shows the histogram of the plotted diameter data of image J software.**

**Figure S3: SEM analysis of PVA/MOF5-NFs concentration (A) shows NFs at a magnification of 2 KX, (B) 10 KX magnification, and (C) shows the histogram of the plotted diameter data of image J software.**

**Figure S4: SEM analysis of PVA/PS10-NFs concentration(A) shows NFs at a magnification of 2 KX, (B) 10 KX magnification, and (C) shows the histogram of the plotted diameter data of image J software.**

**Figure S5: SEM analysis of PVA/PS20-NFs concentration (A) shows NFs at a magnification of 2 KX, (B) 10 KX magnification, and (C) shows the histogram of the plotted diameter data of image J software.**

**Figure S6: SEM analysis of PVA/PS30-NFs concentration (A) shows NFs at a magnification of 2 KX, (B) 10 KX magnification, and (C) shows the histogram of the plotted diameter data of image J software.**

**Figure 7: SEM analysis of PVA/PS40-NFs concentration (A) shows NFs at a magnification of 2 KX, (B) 10 KX magnification, and (C) shows the histogram of the plotted diameter data of image J software.**

**Figure S8: SEM analysis of PVA/MOF5/PS10-NFs concentration (A) show NFs at a magnification of 2 KX, (B) 10 KX magnification, and (C) shows the histogram of the plotted diameter data of image J software.**

**Figure S9: SEM analysis of PVA/MOF5/PS20-NFs parsley concentration (A) show NFs at a magnification of 2 KX, (B) 10 KX magnification, and (C) shows the histogram of the plotted diameter data of image J software.**

**Figure S10: SEM analysis of PVA/MOF5/PS30-NFs concentration (A) show NFs at a magnification of 2 KX, (B) 10 KX magnification, and (C) shows the histogram of the plotted diameter data of image J software.**

**Figure S11: SEM analysis of PVA/MOF5/PS40-NFs concentration (A) show NFs at a magnification of 2 KX, (B) 10 KX magnification, and (C) shows the histogram of the plotted diameter data of image J software.**

**Figure S21: Tensile strength plots of (a) PVA-NF, (b) PVA/MOF1.25-NFs, (c) PVA/MOF2.5-NFs, (d) PVA/MOF5-NFs, (e) PVA/PS10-NFs, (f) PVA/PS20-NFs, (g) PVA/PS30-NFs, (h) PVA/PS40-NFs, (I) PVA/MOF5/PS10-NFs, (j) PVA/MOF5/PS20-NFs, (k) PVA/MOF5/PS30-NFs, (L) PVA/MOF5/PS40-NFs.**

**Table S1. Semi-quantitative evaluation of histological sections**

| **Scale** | **Epithelization** | **PMNL** | **Fibroblasts** | **New vessels** | **Collagen** |
| --- | --- | --- | --- | --- | --- |
| 0 | Thickness of cut edges | absent | absent | absent | absent |
| 1 | Migration of cells (< 50%) | mild ST | mild ST | mild-SCT | minimal-GT |
| 2 | Migration of cells (≥ 50%) | mild DL/GT | mild-GT | mild-GT | mild-GT |
| 3 | Bridging the excision | moderate DL/GT | moderate-GT | moderate-GT | moderate-GT |
| 4 | keratinization | marked DL/GT | marked-GT | marked-GT | marked-GT |

**(ST – surrounding tissue, i.e., tissue out of GT; DL – demarcation line; SCT – subcutaneous tissue; GT – granulation tissue)**
